# Novel Aminoglycoside Resistance Transposons and Transposon-Derived Circular Forms Detected in Carbapenem-Resistant Acinetobacter baumannii Clinical Isolates

**DOI:** 10.1128/AAC.02143-15

**Published:** 2016-02-26

**Authors:** Nabil Karah, Chinmay Kumar Dwibedi, Karin Sjöström, Petra Edquist, Anders Johansson, Sun Nyunt Wai, Bernt Eric Uhlin

**Affiliations:** a Department of Molecular Biology, Laboratory for Molecular Infection Medicine Sweden, and Umeå Centre for Microbial Research, Umeå University, Umeå, Sweden; b Department of Clinical Microbiology and Laboratory for Molecular Infection Medicine Sweden, Umeå University, Umeå, Sweden; c Unit for Antibiotic Resistance and Respiratory Bacterial Infections, Public Health Agency of Sweden, Solna, Sweden

## Abstract

Acinetobacter baumannii has emerged as an important opportunistic pathogen equipped with a growing number of antibiotic resistance genes. Our study investigated the molecular epidemiology and antibiotic resistance features of 28 consecutive carbapenem-resistant clinical isolates of A. baumannii collected throughout Sweden in 2012 and 2013. The isolates mainly belonged to clonal complexes (CCs) with an extensive international distribution, such as CC2 (*n* = 16) and CC25 (*n* = 7). Resistance to carbapenems was related to *bla*_OXA-23_ (20 isolates), *bla*_OXA-24/40-like_ (6 isolates), *bla*_OXA-467_ (1 isolate), and IS*Aba1-bla*_OXA-69_ (1 isolate). Ceftazidime resistance was associated with *bla*_PER-7_ in the CC25 isolates. Two classical point mutations were responsible for resistance to quinolones in all the isolates. Isolates with high levels of resistance to aminoglycosides carried the 16S rRNA methylase *armA* gene. The isolates also carried a variety of genes encoding aminoglycoside-modifying enzymes. Several novel structures involved in aminoglycoside resistance were identified, including Tn*6279*, ΔTn*6279*, Ab-ST3-*aadB*, and different assemblies of Tn*6020* and Tn*aphA6*. Importantly, a number of circular forms related to the IS*26* or IS*Aba125* composite transposons were detected. The frequent occurrence of these circular forms in the populations of several isolates indicates a potential role of these circular forms in the dissemination of antibiotic resistance genes.

## INTRODUCTION

During the last 4 decades, Acinetobacter baumannii has emerged as a major opportunistic pathogen responsible for a wide range of hospital-acquired infections, such as ventilator-associated pneumonia and catheter-related bloodstream infections (1). Multilocus sequence typing (MLST) demonstrated a wide geographical prevalence of particular clonal complexes (CCs) in the global population of A. baumannii (2). According to the Institute Pasteur MLST scheme, CC2, CC1, and, recently, CC25 have been playing a major role in the worldwide dissemination of A. baumannii (2, 3). Worryingly, A. baumannii has shown a remarkable ability to acquire resistance against different classes of antibiotics (1). Resistance to carbapenems is mainly mediated by the production of carbapenem-hydrolyzing enzymes, usually encoded by horizontally imported genes (4). In this regard, a variety of acquired carbapenemase genes have been detected, including the class A (*bla*_GES-14_ and *bla*_KPC_), class B (*bla*_IMP_, *bla*_VIM_, *bla*_SIM-1_, and *bla*_NDM_), and class D (*bla*_OXA-23-like_, *bla*_OXA-24/40-like_, *bla*_OXA-58-like_, *bla*_OXA-104_, *bla*_OXA-143_, *bla*_OXA-164_, and *bla*_OXA-182_) β-lactamases (2, 4, 5). Alternatively, overexpression of the A. baumannii-intrinsic *bla*_OXA-51-like_ gene has also been responsible for carbapenem resistance in A. baumannii (5). The overexpression is usually due to the acquisition of a strong promoter provided by an insertion sequence (IS) element, IS*Aba1*, inserted upstream of the relevant gene (5). Likewise, resistance to ceftazidime has mainly been mediated by the overexpression of another A. baumannii-intrinsic gene, the *bla*_ADC_ (Acinetobacter-derived cephalosporinase) gene, which has also been related to the acquisition of an upstream strong promoter provided by IS*Aba1* or other IS elements (4, 6).

The acquisition of genes encoding aminoglycoside-modifying enzymes (AMEs) has been a main cause of resistance to aminoglycosides in A. baumannii (4). Different AME-encoding genes, such as *aphA1*, *aphA6*, *aphA15*, *aacC1*, *aacC2*, *aacA4*, *aadB*, *aadA1*, and *aadA4*, have been detected in clinical isolates of A. baumannii (2, 4). Many of these genes (for example, *aacC1*, *aacA4*, and *aadA1*) are located on class 1 integrons (7). In contrast, the *aphA1* and *aphA6* genes have always been surrounded by IS elements, forming different composite transposon structures (8, 9). Notably, A. baumannii strains producing the ArmA 16S rRNA methylase have increasingly been reported in the last decade (10). The ArmA enzyme mediates a high level of pan-aminoglycoside resistance, on the basis of the decrease of affinity of the targeted 16S rRNA toward aminoglycosides (11). The *armA* gene has frequently been surrounded by the putative *tnpU* and *tnpD* transposase genes and has repeatedly been found to be plasmid mediated (10, 12). On the other hand, the acquisition of particular chromosomal mutations is the primary mechanism of quinolone resistance in A. baumannii (4). The occurrence of two amino acid substitutions in the quinolone resistance-determining region (QRDR) of the A. baumannii gyrase and topoisomerase IV enzymes has been sufficient for the isolates to obtain a high level of resistance to quinolones (13).

In Sweden, the prevalence of A. baumannii infections has been low and the emergence of multidrug-resistant strains has often been associated with a history of import (14). The ability of A. baumannii to persist in health care settings and the increasing frequency of imports by patients colonized with the bacterium call for enhanced surveillance and attention. This study aimed to characterize the molecular epidemiology and antimicrobial resistance features of a recent collection of carbapenem-resistant A. baumannii clinical isolates collected over a period of 1 and a half years throughout Sweden. In addition, the study expansively investigated the genetic context of particular aminoglycoside resistance genes.

## MATERIALS AND METHODS

### A. baumannii isolates.

The study included 28 consecutive nonduplicate clinical isolates of A. baumannii collected by 8 diagnostic microbiology laboratories throughout Sweden between April 2012 and November 2013 (Table 1). The isolates were selected on the basis of their resistance to carbapenems (imipenem and/or meropenem), according to the clinical breakpoints defined by the European Committee on Antimicrobial Susceptibility Testing (EUCAST; http://www.eucast.org/clinical_breakpoints/). Species identification was first performed using a commercial Microflex system and MALDI Biotyper (version 3.1) software (Bruker Daltonics, Billerica, MA) and then confirmed by detecting the occurrence of *bla*_OXA-51-like_ and partial *rpoB* gene sequences (15, 16).

**TABLE 1:** Epidemiological data and molecular strain typing

Strain	Sample	Date of isolation (day.mo.yr)	City of isolation	Source of imported strain	MLST^*a*^			Pulsotype
					Allelic profile	ST	CC	
A068	No data	04.04.2012	Blekinge	No data	3-3-2-4-7-2-4	ST25	CC25	Ac-02
A069	Feces	24.05.2012	Halland	Thailand	3-3-2-4-7-2-4	ST25	CC25	Sporadic
A070	Tracheal secretion	08.06.2012	Stockholm	No data	27-2-7-2-2-1-2	ST215	CC215	Sporadic
A071	Labia major	02.08.2012	Skåne	No data	2-2-2-2-2-2-2	ST2	CC2	Sporadic
A072	Rectum	28.09.2012	Skåne	No data	2-2-2-2-2-2-2	ST2	CC2	Ac-01
A074	Urine	10.12.2012	Skåne	No data	2-1-2-2-2-1-1	ST636	Singleton	Ac-03
A076	Rectum	04.01.2013	Skåne	No data	1-1-1-1-5-1-1	ST1	CC1	Sporadic
A077	Rectum	10.01.2013	Skåne	No data	2-2-2-2-2-2-2	ST2	CC2	Sporadic
A078	Tracheal secretion	05.02.2013	Våstra Götaland	No data	1-3-10-1-4-4-4	ST23	CC10	Sporadic
A079	No data	07.02.2013	Skåne	No data	2-2-2-2-2-2-2	ST2	CC2	Ac-01
A080	Tracheal secretion	11.03.2013	Skåne	No data	2-2-2-2-2-2-2	ST2	CC2	Ac-01
A082	Wound	12.03.2013	Östergötland	No data	1-1-1-1-5-1-1	ST1	CC1	Sporadic
A084	Groin	26.03.2013	Östergötland	Iraq	2-2-2-2-2-2-2	ST2	CC2	Ac-01
A085	Rectum	02.04.2013	Östergötland	Israel	3-3-2-2-3-1-3	ST3	CC3	Sporadic
A086	Rectum	16.04.2013	Skåne	No data	2-2-2-2-2-2-2	ST2	CC2	Ac-01
A087	Urine	23.04.2013	Västra Götaland	No data	2-2-2-2-2-2-2	ST2	CC2	Sporadic
A089	No data	08.05.2013	Västernorrland	Thailand	2-2-2-2-2-2-2	ST2	CC2	Ac-01
A091	Feces	31.05.2013	Varmland	Italy	2-2-2-2-2-2-2	ST2	CC2	Sporadic
A092	Blood	01.08.2013	Östergötland	No data	3-3-2-4-7-2-4	ST25	CC25	Ac-02
A093	No data	02.08.2013	Östergötland	No data	3-3-2-4-7-2-4	ST25	CC25	Ac-02
A094	Feces	02.08.2013	Östergötland	No data	3-3-2-4-7-2-4	ST25	CC25	Ac-02
A095	Feces	14.08.2013	Stockholm	No data	2-2-2-2-2-2-2	ST2	CC2	Sporadic
A096	Thorax	16.08.2013	Östergötland	No data	3-3-2-4-7-2-4	ST25	CC25	Ac-02
A097	Nose	21.08.2013	Östergötland	No data	3-3-2-4-7-2-4	ST25	CC25	Ac-02
A099	Bronchial secretion	11.10.2013	Östergötland	No data	5-2-4-1-3-3-4	ST85	CC85	Sporadic
A100	No data	17.10.2013	Skåne	No data	1-1-1-1-5-1-1	ST1	CC1	Sporadic
A101	Decubitus sore	24.10.2013	Östergötland	No data	2-2-2-2-2-2-2	ST2	CC2	Ac-01
A105	Bronchial secretion	21.11.2013	Stockholm	No data	2-1-2-2-2-1-1	ST636	Singleton	Ac-03

a MLST, multilocus sequence typing (performed according to the Institute Pasteur scheme); ST, sequence type; CC, clonal complex.

### Antimicrobial susceptibility testing.

The MICs of cefotaxime, ceftazidime, imipenem, meropenem, ciprofloxacin, and colistin were determined using Etest (bioMérieux SA, Marcy l'Etoile, France) according to the manufacturer's instructions. The diameters of the zones of inhibition for tobramycin, gentamicin, and amikacin were determined by the agar disk diffusion method using disks from Oxoid (Basingstoke, United Kingdom) and following the guidelines of EUCAST (http://www.eucast.org/antimicrobial_susceptibility_testing/). Susceptibility patterns were interpreted according to the clinical breakpoints defined by EUCAST (http://www.eucast.org/clinical_breakpoints/) for all antibiotics except cefotaxime and ceftazidime, for which the Clinical and Laboratory Standards Institute (CLSI) breakpoints were used (CLSI document M100-S24, 2014 [17]). Isolates showing resistance to ≥3 classes of antibiotics were defined to be multidrug resistant (18).

### Genome sequencing and sequence analysis.

Whole DNA genomes were extracted by use of a DNeasy blood and tissue kit (Qiagen, Sollentuna, Sweden) and quantified using a Qubit double-stranded DNA BR assay system (Life Technologies, Stockholm, Sweden). An indexed paired-end library was prepared for all the isolates using a Nextera XT DNA sample preparation kit (Illumina, San Diego, CA, USA), a Nextera XT index kit (Illumina, San Diego, CA, USA), and an Agencourt AMPure XP system (Beckman Coulter, Bromma, Sweden). The library was sequenced using a MiSeq reagent kit (v3) and an Illumina MiSeq system (Illumina, San Diego, CA, USA). All steps of DNA preparation, library construction, and genome sequencing were done according to the manufacturers' instructions. Sequence data were assembled and analyzed using the CLC genomics workbench (v7.0.4; CLC bio, Aarhus, Denmark).

### Molecular epidemiology.

The isolates were assigned to sequence types (STs) using the MLST function provided online by the Center for Genomic Epidemiology (http://www.genomicepidemiology.org/) in Denmark (19). The assignment was performed according to the Institute Pasteur's scheme (3). In order to allocate the isolates into CCs, the BURST algorithm, integrated in the Institute Pasteur's MLST website (http://www.pasteur.fr/mlst), was applied to all the allelic profiles in the database under a stringent grouping parameter (profiles that matched the profile of any other member of the group at 6 loci were defined to be a group). Pulsed-field gel electrophoresis (PFGE) was performed using ApaI-digested genomic DNA, as previously described (20). DNA fragments were separated in a CHEF-DRII apparatus (Bio-Rad, Marne LA Coquette, France) at 6 V/cm and 14°C for 20 h with switch times ranging from 5 s to 30 s. Similarities among the PFGE patterns were calculated by the Dice coefficient method (with an optimization of 1% and a position tolerance of 1%) using BioNumerics software (v7.1; Applied Maths, Ghent, Belgium). A PFGE type was defined by a cluster of isolates showing ≥90% similarity or less than 3 band differences (21).

In addition, a phylogenetic analysis based on alignments of the whole-genome single nucleotide polymorphism (SNP) concatenations was performed. The genomes were assembled using ABySS (v2.0; Canada's Michael Smith Genome Sciences Centre, Vancouver, Canada), a *de novo* assembler designed for short-read sequence data (22). Pairwise alignments were performed using the progressiveMauve algorithm (v12; http://darlinglab.org/mauve/user-guide/progressivemauve.html) and A. baumannii ATCC 17978 as an index strain. Then, all the duo alignments were merged into one multiple-sequence alignment. Nucleotide distances between genomes were calculated using MEGA (v5.13; http://www.megasoftware.net/) and SplitsTree (http://www.splitstree.org/) software. A. baumannii strains ATCC 17978 (sporadic), AYE (CC1), ACICU (CC2), and SDF (sporadic, isolated from a human body louse) were included as reference strains. SplitsTree was also used to generate a phylogenetic NeighborNet graph.

### Detection of antibiotic resistance genes and genetic elements.

ResFinder, a search engine hosted on the website of the Center for Genomic Epidemiology (http://www.genomicepidemiology.org/) in Denmark, was used to detect the presence of acquired antimicrobial resistance genes (23). The ResFinder database includes more than 1,700 variants of antibiotic resistance genes (last accessed in March 2015). IS elements were identified using the ISfinder application (24). Plasmids were typed according to the A. baumannii PCR-based replicon typing (AB-PBRT) scheme (25). Plasmid typing was performed using *in silico* PCR assays followed by sequence analysis of the positive amplicons. Comparative sequence analysis was performed in order to detect the occurrence of amino acid replacements in the QRDRs of GyrA and ParC, using, respectively, the GenBank (GB) sequences of GyrA (GB accession number CAA57655) and ParC (GB accession number CAA65085) produced by quinolone-susceptible isolates as controls (13, 26).

**FIG 1:**
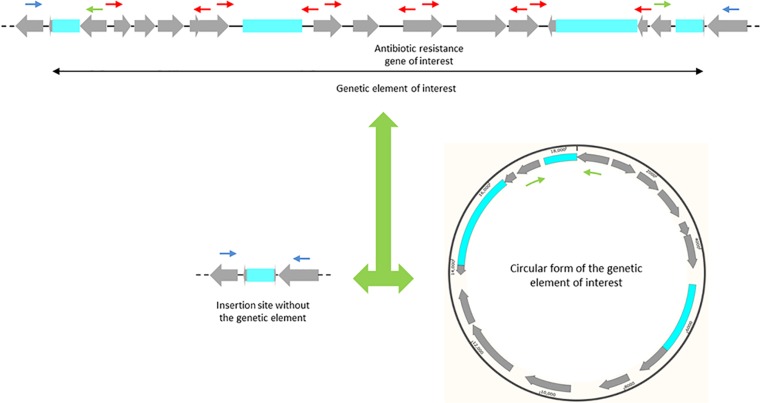
Illustrative positioning of the primers. Gray arrows, coding regions, with the arrowhead indicating the direction of transcription; blue boxes, insertion sequence elements; black double-headed arrow, a presumed genetic element carrying an antibiotic resistance gene of interest. The figure shows an inserted genetic element surrounded by two peripheral copies of an IS element (top) coexisting with a circular form with only one copy of these IS elements (bottom right) and an insertion site with the second copy (bottom left). The genetic context was verified using a number of internal primers (red arrows). Insertion of the genetic element was detected by PCR assays and sequencing using an outwards internal and an inwards external primer (one green arrow and one blue arrow). The occurrence of circular forms was detected by PCR assays and sequencing using peripheral outwards internal primers (the two green arrows). A deficiency of the genetic element in part of the population of each strain was investigated using inwards external primers (the two blue arrows).

PCR amplification and Sanger sequencing of the amplicons were used to determine the order and orientation of some contigs carrying resistance determinants and to fill in gaps between these contigs, as previously described (27). In addition, the genetic context of particular antibiotic resistance genes and genetic elements was determined by PCR assays and sequencing using more than 120 inwards and outwards, internal and external primers (see Table S1 in the supplemental material). The occurrence of circular forms was detected by PCR assays and sequencing using the outwards internal primers, whereas misplacement of the genetic element in part of the population of each strain was investigated using the inwards external primers (Fig. 1). One thermal cycling program consisting of an initial denaturation at 94°C for 5 min followed by 30 cycles of amplification (denaturation at 95°C for 20 s, annealing at 56°C for 20 s, and extension at 72°C for 2.5 min) and a final extension at 72°C for 8 min was used for all the PCRs.

### Nucleotide sequence accession numbers.

Draft genome sequences of all the isolates were deposited in the DDBJ/EMBL/GenBank database under accession numbers SRS925054 (strain A068), SRS926091 and SRS926093 (strains A069 and A070, respectively), SRS927389 to SRS927398 (strains A071 to A084, respectively), SRS927399 and SRS927400 (strains A086 and A087, respectively), SRS927401 (strain A085), SRS927402 to SRS927412 (strains A089 to A101, respectively), and SRS979539 (strain A105). In addition, novel sequences of particular genetic elements were manually annotated and deposited in the DDBJ/EMBL/GenBank database under the following accession numbers: KR535992 (plasmid pA105-1 carrying Tn*aphA6*), KR535993 (plasmid pA105-2 carrying IS*Aba31* and *bla*_OXA-72_), KP881233 to KP881241 (*bla*_ADC-73_ to *bla*_ADC-81_, respectively, representing novel variants of *bla*_ADC_), KT354505 (*bla*_OXA-467_, representing a novel variant of *bla*_OXA-58-like_), KT317075 (Tn*6279* inserted in the *orf*_HPA2_ gene of strain A071, representing a subpopulation of this strain), KT317076 (Tn*1548*-like-1 inserted in the *orf*_HPA2_ gene of strain A071, representing a second subpopulation of this strain), KT317077 (IS*26* inserted in the *orf*_HPA2_ gene of strain A071, representing a third subpopulation of this strain), KT354506 (Tn*6279*-like chromosomally inserted in strain A070), KT354507 (ΔTn*6279* chromosomally inserted in strain A072), KT317078 to KT317084 (circular forms of Tn*6279*, Tn*1548*-like-1, Tn*6020b-1*, Tn*6020b-2*, Tn*6020a-1*, Tn*6020a-2*, and Tn*6020a-3*, respectively), KT317085 (Tn*1548*-like-2 inserted in a plasmid), KT317086 (circular form of Tn*1548*-like-2), and KT317087 (circular form of Tn*aphA6a*). The novel sequences of ST636, *bla*_OXA-467_, and IS*Aba31* were also deposited in the locus/sequence definitions database of the Institut Pasteur Acinetobacter MLST system (http://pubmlst.org/abaumannii/), Lahey Clinic databases of β-lactamases (http://www.lahey.org/Studies/), and the ISfinder database (www-is.biotoul.fr), respectively. The novel Tn*6279* transposon was numbered by the curators of the Tn Number Registry (http://www.ucl.ac.uk/eastman/research/departments/microbial-diseases/tn) (28).

## RESULTS

### Species identification and antimicrobial susceptibility patterns.

Assignment of all the isolates to the A. baumannii species was confirmed by biochemical characterization, presence of the *bla*_OXA-51-like_ gene, and partial *rpoB* sequence analysis (see Fig. S1 in the supplemental material). Resistance to meropenem or imipenem was confirmed for all the isolates. However, one isolate (A082) was intermediate to imipenem, while another isolate (A099) was intermediate to meropenem (Table 2). All the isolates showed high levels of resistance (MICs ≥ 32 mg/liter) to ciprofloxacin. Twenty-seven isolates were resistant to cefotaxime and ceftazidime, while only one isolate (A094) was intermediate to cefotaxime and fully susceptible to ceftazidime. Sixteen isolates showed high levels of resistance (diameter of zone of inhibition, 6 mm) to all the three aminoglycosides. The other 12 isolates revealed variable levels of resistance to one or more of the aminoglycosides. All the isolates were defined to be multidrug resistant (18). Colistin was the only antibiotic to which all the isolates were susceptible.

**TABLE 2:** Antimicrobial susceptibility patterns^*a*^

Strain	Susceptibility by Etest (MIC [mg/liter])						Susceptibility by agar disc diffusion (zone diam [mm])		
	MEM	IPM	CIP	CTX	CAZ	CST	TOB10	GEN10	AMK30
A068	R (16)	R (32)	R (>32)	R (>256)	R (>256)	S (0,25)	R (6)	R (6)	R (6)
A069	R (32)	R (32)	R (32)	R (256)	R (256)	S (0.125)	R (6)	R (6)	R (6)
A070	R (32)	R (32)	R (32)	R (256)	R (256)	S (0.25)	R (6)	R (6)	R (6)
A071	R (32)	R (32)	R (32)	R (256)	R (256)	S (0.5)	R (6)	R (6)	R (6)
A072	R (32)	R (32)	R (32)	R (256)	R (256)	S (0.25)	R (6)	R (6)	R (6)
A074	R (32)	R (32)	R (32)	R (256)	R (256)	S (0.25)	S (17)	R (6)	R (6)
A076	R (32)	R (32)	R (32)	R (256)	R (256)	S (0.25)	R (11)	R (6)	S (21)
A077	R (>32)	R (>32)	R (>32)	R (>256)	R (>256)	S (0.25)	S (18)	R (6)	S (20)
A078	R (>32)	R (>32)	R (>32)	R (>256)	R (>256)	S (0.25)	R (6)	R (6)	R (9)
A079	R (>32)	R (>32)	R (>32)	R (>256)	R (>256)	S (0.125)	R (6)	R (6)	R (6)
A080	R (>32)	R (>32)	R (>32)	R (>256)	R (>256)	S (0.25)	R (6)	R (6)	R (6)
A082	R (16)	I (8)	R (>32)	R (>256)	R (>256)	S (0.25)	R (12)	R (6)	R (9)
A084	R (>32)	R (>32)	R (>32)	R (>256)	R (>256)	S (0.5)	S (19)	R (8)	R (8)
A085	R (>32)	R (>32)	R (>32)	R (>256)	R (256)	S (0.5)	R (14)	R (10)	I (15)
A086	R (32)	R (32)	R (32)	R (256)	R (256)	S (0.125)	R (6)	R (6)	R (6)
A087	R (32)	R (32)	R (32)	R (256)	R (256)	S (0.125)	S (18)	R (6)	R (6)
A089	R (32)	R (32)	R (32)	R (256)	R (256)	S (0.125)	R (6)	R (6)	R (6)
A091	R (32)	R (32)	R (32)	R (256)	R (256)	S (0.25)	R (6)	R (6)	R (6)
A092	R (32)	R (32)	R (32)	R (256)	R (256)	S (1)	R (6)	R (6)	R (6)
A093	R (32)	R (32)	R (32)	R (256)	R (256)	S (0.25)	R (6)	R (6)	R (6)
A094	R (32)	R (32)	R (32)	I (32)	S (8)	S (0.25)	R (6)	R (6)	R (6)
A095	R (32)	R (32)	R (32)	R (256)	R (256)	S (0.5)	R (6)	R (6)	R (6)
A096	R (32)	R (32)	R (32)	R (256)	R (256)	S (0.25)	R (6)	R (6)	R (6)
A097	R (32)	R (32)	R (32)	R (256)	R (256)	S (0.25)	R (6)	R (6)	R (6)
A099	I (8)	R (16)	R (32)	R (256)	R (32)	S (0.125)	S (18)	R (8)	R (10)
A100	R (32)	R (32)	R (32)	R (256)	R (256)	S (0.125)	R (7)	R (6)	R (8)
A101	R (32)	R (32)	R (32)	R (256)	R (256)	S (0.125)	S (19)	S (19)	R (10)
A105	R (32)	R (32)	R (32)	R (256)	R (256)	S (0.125)	S (19)	R (6)	R (8)

a MEM, meropenem; IPM, imipenem; CIP, ciprofloxacin; CTX, cefotaxime; CAZ, ceftazidime; CST, colistin; TOB10, tobramycin at 10 mg/liter; GEN10, gentamicin at 10 mg/liter; AMK30, amikacin at 30 mg/liter; R, resistant; I, intermediate; S, susceptible. The results were interpreted according to the following clinical breakpoints, as defined by EUCAST (http://www.eucast.org/clinical_breakpoints/) and/or according to the Clinical and Laboratory Standards Institute breakpoints (CLSI document M100-S24, 2014 [17]): for meropenem and imipenem, susceptible was an MIC of ≤2 mg/liter and resistant was an MIC of >8 mg/liter; for ciprofloxacin, susceptible was an MIC of ≤1 mg/liter and resistant was an MIC of >1 mg/liter; for cefotaxime, susceptible was an MIC of ≤8 mg/liter, intermediate was an MIC of 16 to 32 mg/liter, and resistant was an MIC of ≥64 mg/liter; for ceftazidime, susceptible was an MIC of ≤8 mg/liter, intermediate was an MIC of 16 mg/liter, and resistant was an MIC of ≥32 mg/liter; for colistin, susceptible was an MIC of ≤2 mg/liter and resistant was an MIC of >2 mg/liter; for tobramycin at 10 mg/liter, susceptible was an MIC of ≥17 mg/liter and resistant was an MIC of <17 mg/liter; for gentamicin at 10 mg/liter, susceptible was an MIC of ≥17 mg/liter and resistant was an MIC of <17 mg/liter; and for amikacin at 30 mg/liter, susceptible was an MIC of ≥18 mg/liter and resistant was an MIC of <15 mg/liter).

### Molecular epidemiology.

**FIG 2:**
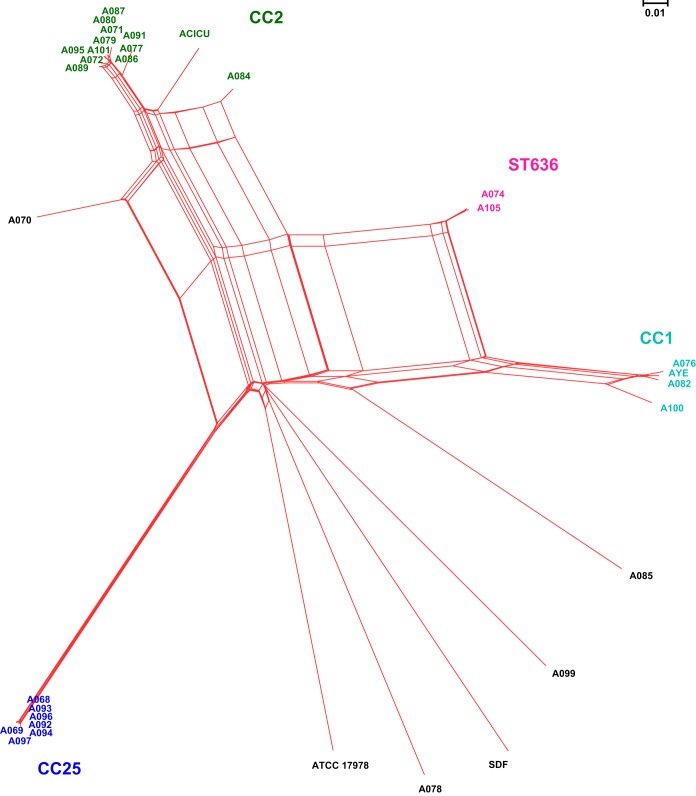
Whole-genome SNP-based phylogenetic network (constructed using NeighborNet graph). The NeighborNet graph was constructed using SplitsTree (http://www.splitstree.org/) on the basis of the alignments of the whole-genome SNP concatenations of 28 Acinetobacter baumannii isolates. Strains ATCC 17978 (sporadic), AYE (CC1), ACICU (CC2), and SDF (sporadic, isolated from a human body louse) were included as reference strains. The corresponding sequence type (ST) or clonal complex (CC) was added next to the four main clades.

The isolates were assigned to ST2/CC2 (*n* = 12), ST25/CC25 (*n* = 7), ST1/CC1 (*n* = 3), ST636/singleton (*n* = 2), ST215/CC215 (*n* = 1), ST23/CC10 (*n* = 1), ST3/CC3 (*n* = 1), and ST85/CC85 (*n* = 1) (Table 1). In general, the CCs showed a robust association with particular variants of the intrinsic *bla*_OXA-51-like_ and *bla*_ADC_ genes. For instance, all the CC25 isolates carried the *bla*_OXA-64_ (*n* = 7) and *bla*_ADC-26_ (*n* = 7) variants, whereas *bla*_OXA-66_ (*n* = 13), *bla*_ADC-73_ (*n* = 7), and *bla*_ADC-30_ (*n* = 6) were exclusively present in isolates from CC2 or CC215. Whole-genome SNP-based phylogenetic analysis identified 4 clades corresponding to CC1, CC2, CC25, and ST636, according to the MLST nomenclature (Fig. 2). The analysis also detected several conflicting phylogenetic signals, represented by reticulate topologies in SplitsTree, possibly related to the occurrence of frequent recombination events. PFGE allocated 15 of the isolates into three groups, namely, Ac-01 (*n* = 7), Ac-02 (*n* = 6), and Ac-03 (*n* = 2), consistent with CC2, CC25, and ST236, respectively (see Fig. S2 in the supplemental material). The remaining 13 isolates had unique PFGE patterns. A linkage between Ac-01 and Ac-02 and two particular laboratories, which may indicate a local spread of outbreak strains, was observed (Table 1).

### Antibiotic resistance mechanisms.

Resistance to carbapenems was mediated by *bla*_OXA-23_ in 20 isolates, *bla*_OXA-24/40_ or *bla*_OXA-72_ in 6 isolates, *bla*_OXA-467 (OXA-58-like)_ in 1 isolate, and IS*Aba1-bla*_OXA-69_ in 1 isolate (Table 3). Resistance to third-generation cephalosporins was associated with IS*Aba1-bla*_ADC-like_ (19 isolates), *bla*_PER-7_ (6 isolates), IS*Aba125-bla*_ADC-81_ (1 isolate), and IS*6*-family-*bla*_ADC-77_ (1 isolate). It is important to note that the last isolate (A078) carried two *bla*_ADC_ genes; one of them (*bla*_ADC-76_) was present at the typical chromosomal position for A. baumannii-intrinsic *bla*_ADC_ genes, while the other one (*bla*_ADC-77_) was located elsewhere and surrounded by two copies of IS*6*-family. Resistance to quinolones was due to two amino acid substitutions in the QRDRs of GyrA (S83L) and ParC (S80L) in all the isolates. All the 16 isolates with high levels of resistance to amikacin, gentamicin, and tobramycin carried the *armA* gene (Table 4). In addition, the *aacA4*, *aacC1*, *aacC2*, *aadA1*, *aadB*, *aphA1*, and *aphA6* aminoglycoside resistance genes were detected in 4, 9, 7, 10, 2, 16, and 11 isolates, respectively. The isolates also carried a number of streptomycin, macrolide, phenicol, rifampin, or sulfonamide resistance genes (see Tables S2 and S3 in the supplemental material).

**TABLE 3:** β-Lactam resistance genes and genetic context

Strain	Intrinsic genes		Acquired genes	
	*bla*_ADC_	*bla*_OXA-51-like_	*bla*_OXA_ (genetic context)	Other(s)
A068	*bla*_ADC-26_	*bla*_OXA-64_	*bla*_OXA-23_ (Tn*2006*)	*bla*_PER-7_
A069	*bla*_ADC-26_	*bla*_OXA-64_	*bla*_OXA-23_ (Tn*2006*)	*bla*_PER-7_
A070	IS*Aba1*, *bla*_ADC-30_	*bla*_OXA-66_	*bla*_OXA-23_ (Tn*2006*)	*bla*_TEM-1D_
A071	IS*Aba1*, *bla*_ADC-30_	*bla*_OXA-66_	*bla*_OXA-23_ (Tn*2009*)	
A072	IS*Aba1*, *bla*_ADC-73_	*bla*_OXA-66_	*bla*_OXA-23_ (Tn*2006*)	*bla*_TEM-1D_
A074	IS*Aba1*, *bla*_ADC-74_	*bla*_OXA-66_	XerC/XerD-like, IS*Aba31*, *bla*_OXA-72_, XerC/XerD-like	
A076	IS*Aba1*, *bla*_ADC-75_	IS*Aba1*, *bla*_OXA-69_		*bla*_TEM-1D_
A077	IS*Aba1*, *bla*_ADC-30_	*bla*_OXA-66_	XerC/XerD-like, *bla*_OXA-24/40_, XerC/XerD-like	
A078	*bla*_ADC-76_	*bla*_OXA-68_	XerC/XerD-like, IS*Aba31*, *bla*_OXA-72_, XerC/XerD-like	IS*6*-family, *bla*_ADC-77_
A079	IS*Aba1*, *bla*_ADC-73_	*bla*_OXA-66_	*bla*_OXA-23_ (Tn*2006*)	*bla*_TEM-1D_
A080	IS*Aba1*, *bla*_ADC-73_	*bla*_OXA-66_	*bla*_OXA-23_ (Tn*2006*)	*bla*_TEM-1D_
A082	IS*Aba1*, *bla*_ADC-78_	*bla*_OXA-69_	*bla*_OXA-23_ (Tn*2006*)	*bla*_TEM-1D_
A084	IS*Aba1*, *bla*_ADC-30_	*bla*_OXA-66_	*bla*_OXA-23_ (Tn*2006*)	*bla*_TEM-1D_
A085	IS*Aba1*, *bla*_ADC-79_	*bla*_OXA-71_	*bla*_OXA-23_ (Tn*2006*)	
A086	IS*Aba1*, *bla*_ADC-73_	*bla*_OXA-66_	*bla*_OXA-23_ (Tn*2006*)	*bla*_TEM-1D_
A087	IS*Aba1*, *bla*_ADC-30_	*bla*_OXA-66_	XerC/XerD-like, *bla*_OXA-24/40_, XerC/XerD-like	
A089	IS*Aba1*, *bla*_ADC-73_	*bla*_OXA-66_	*bla*_OXA-23_ (Tn*2006*)	
A091	IS*Aba1*, *bla*_ADC-30_, IS*Aba1*	*bla*_OXA-66_	*bla*_OXA-23_ (Tn*2006*)	
A092	*bla*_ADC-26_	*bla*_OXA-64_	*bla*_OXA-23_ (Tn*2006*)	*bla*_PER-7_
A093	*bla*_ADC-26_	*bla*_OXA-64_	*bla*_OXA-23_ (Tn*2006*)	*bla*_PER-7_
A094	*bla*_ADC-26_	*bla*_OXA-64_	*bla*_OXA-23_ (Tn*2006*)	
A095	IS*Aba1*, *bla*_ADC-73_	*bla*_OXA-66_	*bla*_OXA-23_ (Tn*2006*)	*bla*_TEM-1D_
A096	*bla*_ADC-26_	*bla*_OXA-64_	*bla*_OXA-23_ (Tn*2006*)	*bla*_PER-7_
A097	*bla*_ADC-26_	*bla*_OXA-64_	*bla*_OXA-23_ (Tn*2006*)	*bla*_PER-7_
A099	IS*Aba1*, *bla*_ADC-80_	*bla*_OXA-94_	IS*Aba2*, Δ*ISAba3*, *bla*_OXA-467_, IS*Aba3*	*bla*_TEM-1D_, *bla*_CARB-8_
A100	IS*Aba125*, *bla*_ADC-81_	*bla*_OXA-92_	XerC/XerD-like, IS*Aba31*, *bla*_OXA-72_, XerC/XerD-like	*bla*_TEM-1D_
A101	IS*Aba1*, *bla*_ADC-73_	*bla*_OXA-66_	*bla*_OXA-23_ (Tn*2006*)	*bla*_TEM-1D_
A105	IS*Aba1*, *bla*_ADC-74_	*bla*_OXA-66_	XerC/XerD-like, IS*Aba31*, *bla*_OXA-72_, XerC/XerD-like	

**TABLE 4:** Aminoglycoside resistance genes and genetic context

Strain	16S rRNA methylase gene	Aminoglycoside-modifying enzyme(s) (genetic context)		
		*aphA1*	*aphA6*	Other
A068	*armA*			
A069	*armA*			IS*6*-family, ΔIS*26*, *aacC2*, ΔIS*kpn11*
A070	*armA*	*aphA1b* (Tn*6020a-2*), Δ*aphA1b* (Tn*6020b-2*)		*intI*, *aacC1*, *orfP*, *orfP*, *orfQ*, *aadA1*; Δ*intI1*, *aacA4*, *catB8*, *aadA1*
A071	*armA*	*aphA1b* (Tn*6020b-1*)		Δ*intI1*, *aacA4*, *catB8*, *aadA1*
A072	*armA*	*aphA1b* (Tn*6020a-1*)		
A074		*aphA1b* (Tn*6020a-2*)	*aphA6* (Tn*aphA6*)^*a*^	*intI1*, *aacC1*, *orfP*, *orfQ*, *aadA1* (1st copy); *intI1*, *aacC1*, *orfP*, *orfQ*, *aadA1* (2nd copy)
A076		*aphA1b* (Tn*6020a-3*)		*intI1*, *aacC1*, *orfP*, *orfQ*, *aadA1*
A077				*intI1*, *aacC1*, *orfP*, *orfP*, Δ*orfQ*
A078			*aphA6* (Tn*aphA6*)	*aadB*
A079	*armA*	*aphA1b* (Tn*6020a-1*)		
A080	*armA*	*aphA1b* (Tn*6020a-1*)		
A082		*aphA1b* (Tn*6020a-2*)		*intI1*, *aacC1*, *orfP*, *orfQ*, *aadA1*; IS*6*-family, ΔIS*Aba14*, *aacC2*, *abc*-ATPase, *orf2*, *orf3*, ΔIS*Cfr1*, IS*6*-family
A084		*aphA1b* (Tn*6020a-1*)		*intI*, *aacC1*, *orfP*, *orfP*, *orfQ*, *aadA1*
A085				*intI1*, *aadB*
A086	*armA*	*aphA1b* (Tn*6020a-1*)		
A087				*intI1*, *aacC1*, *orfP*, *orfP*, Δ*orfQ*
A089	*armA*			
A091	*armA*	*aphA1b* (Tn*6020b-1*)		Δ*intI1*, *aacA4*, *catB8*, *aadA1*
A092	*armA*		*aphA6* (Tn*aphA6*)^*b*^	IS*6*-family, ΔIS*26*, *aacC2*, ΔIS*kpn11*
A093	*armA*			IS*6*-family, ΔIS*26*, *aacC2*, ΔIS*kpn11*
A094	*armA*		*aphA6* (Tn*aphA6*)^*b*^	IS*6*-family, ΔIS*26*, *aacC2*, ΔIS*kpn11*
A095	*armA*	*aphA1b* (Tn*6020a-1*)	*aphA6* (Tn*aphA6*)^*b*^	
A096	*armA*		*aphA6* (Tn*aphA6*)^*b*^	IS*6*-family, ΔIS*26*, *aacC2*, ΔIS*kpn11*
A097	*armA*		*aphA6* (Tn*aphA6*)^*b*^	IS*6*-family, ΔIS*26*, *aacC2*, ΔIS*kpn11*
A099		*aphA1b* (Tn*6020a-2*)	*aphA6* (Tn*aphA6*)^*b*^	*intI1*, *aacC1*, *orfP*, *orfP*, *orfQ*, *aadA1*
A100		*aphA1b* (Tn*6020a-2*)	*aphA6* (Tn*aphA6*)	Δ*intI1*, *aacA4*, *aacC1*, *orfP*, *orfQ*, *aadA1*
A101		*aphA1b* (Tn*6020a-1*)	*aphA6* (Tn*aphA6*-like)^*b*^^,^^*c*^	
A105		*aphA1b* (Tn*6020a-2*)	*aphA6* (Tn*aphA6*)^*a*^	*intI1*, *aacC1*, *orfP*, *orfP*, *orfQ*, *aadA1*; *intI1*, *aacC1*, *orfP*, *orfQ*, *aadA1*

a Inserted at a novel location on an *aci6*-carrying plasmid similar to pAb-G7-2.

b Inserted at the same location at which it is inserted in pAb-G7-2.

c Associated with a deletion of 1,165 bp in the host plasmid.

### Genetic surroundings of antibiotic resistance genes.

The *bla*_OXA-23_ gene was located in Tn*2006* in 19/20 of the *bla*_OXA-23_-positive isolates (Table 3). Tn*2006* was carried on AbaR4-like resistance islands interrupting the *comM* gene in many of the isolates, including all the CC25 isolates (data not shown). Only one isolate (A071) carried the *bla*_OXA-23_ gene on a Tn*2009* transposon, inserted chromosomally at the same position where it is located in A. baumannii strain MDR-ZJ06 (GB accession number CP001937) (29). The *bla*_OXA-467_ gene was surrounded by upstream and downstream IS*Aba3* elements, of which the upstream copy was interrupted by another IS element, namely, IS*Aba2* (Table 3). A similar genetic structure was found in A. baumannii isolate MAD (GB accession number AY665723) and in several A. baumannii plasmids, such as pOUR, pABNA1, pABNA2, and pACICU1 (GB accession numbers NG_036424, NG_036787, NG_036788, and CP000864, respectively) (30). A linkage between *bla*_OXA-467_ and a contig carrying the AciX replication gene, representing GR10 of the A. baumannii plasmids, was detected (25).

**FIG 3:**
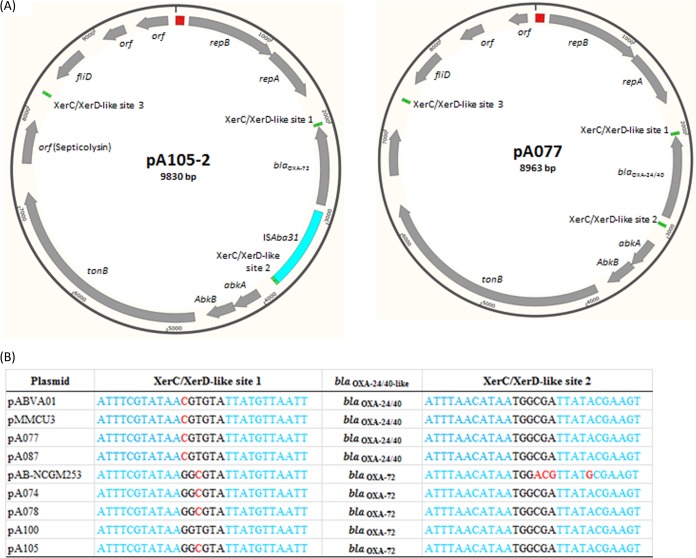
Genetic context of *bla*_OXA-24/40_ and *bla*_OXA-72_. (A) Genetic structures of plasmids pA105-2 and pA077. Gray arrows, coding regions, with the arrowhead indicating the direction of transcription; blue, red, and green boxes, IS*Aba31*, iterons, and XerC/XerD-like sites, respectively. (B) Nucleotide sequence of the XerC/XerD-like sites surrounding the *bla*_OXA-24/40_ and *bla*_OXA-72_ genes. Blue, XerC/XerD-like sequences; red, polymorphisms.

All the *bla*_OXA-24/40_ and *bla*_OXA-72_ genes were flanked by XerC/XerD-like sites and were located on GR2 (Aci1)-type plasmids (see Tables S4 and S5 in the supplemental material) (25). The *bla*_OXA-24/40_-carrying plasmids showed >99% identity to pABVA01 and pMMCU3 (GB accession numbers FM210331 and GQ904227, respectively), which were recovered from A. baumannii strains detected in Italy and Spain (31, 32), whereas the *bla*_OXA-72_-carrying plasmids were more related to pAB-NCGM253 (GB accession number AB823544), a plasmid carried by A. baumannii strains from Japan (33). Interestingly, the replication genes of the *bla*_OXA-24/40_- and *bla*_OXA-72_-carrying plasmids shared only 86% nucleotide sequence identity to each other (see Fig. S3 in the supplemental material). A novel IS element was inserted upstream of *bla*_OXA-72_ in the four *bla*_OXA-72_-positive isolates, although they were recovered in 3 different cities in Sweden (Table 3 and Fig. 3). The new IS, designated IS*Aba31*, was 858 bp long and possessed only one gene encoding a transposase of 248 amino acids with putative transposase of the IS*4*/*5* family and transposase DDE domains (pfam13340 and pfam13586, respectively). IS*Aba31* has imperfect terminal inverted repeats of 16 bp (GGGTGTGTTGACACTT and AAGTGTCAACAGACCC). The insertion of IS*Aba31* generated a 2-bp AT duplication in the target site. Three additional copies of IS*Aba31* were present in the genome of one of our isolates (isolate A100). IS*Aba31* was not present in the genome of the other isolates, except for A099, which carried four copies of an IS*Aba31*-like element (data not shown).

**FIG 4:**
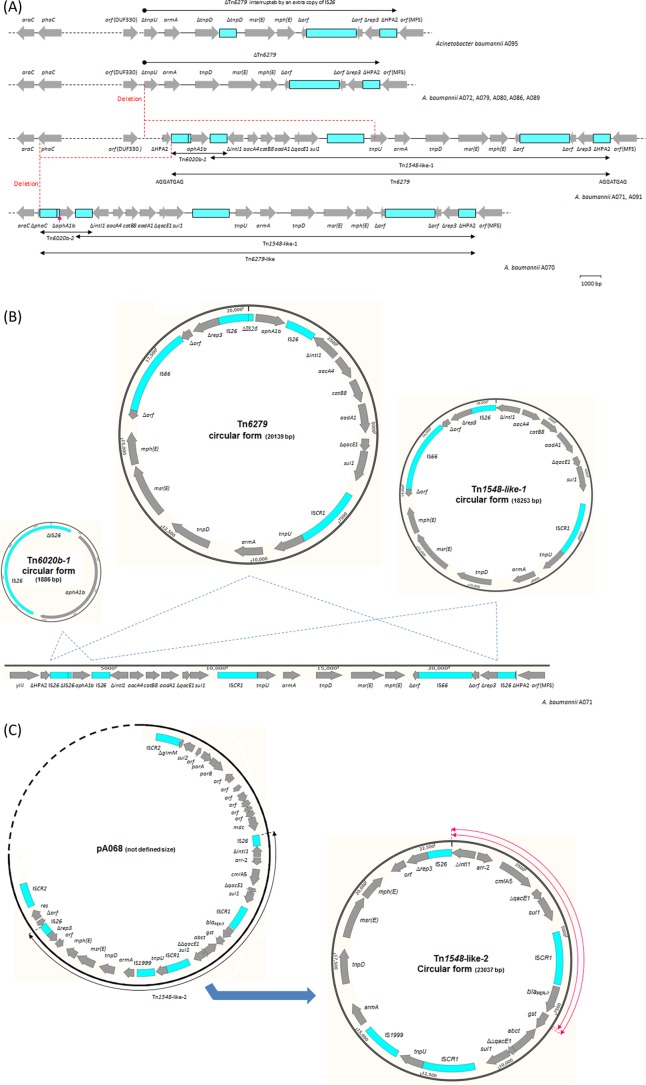
Genetic context of the *armA*-positive elements. (A) Genetic structure and site of chromosomal acquisition of Tn*6279* and ΔTn*6279*. (B) Co-occurrence of circular forms derived from Tn*6279*, Tn*1548*-like-1, and Tn*6020b-1* in Acinetobacter baumannii isolate A071. (C) Plasmid insertion and co-occurrence of a circular form of Tn*1584*-like-2 in A. baumannii isolate A068. Blue labeled arrows, genes, with the arrowhead indicating the direction of transcription; blue boxes, insertion sequence elements; red arrow in panel A, the site of a deletion of 160 bp, making the only difference between Tn*6020b-1* and -*2*; red circular arrows in panel C, the locations of internal deletions of 7,984 and 7,907 bp, resulting in the formation of circular forms derived from Tn*1584*-like-2-Δ1 and -Δ2, respectively.

The *armA* gene was located on the chromosome in nine isolates (ST2 and ST215) and on plasmids in seven isolates (ST25) (Table 5). All the nine chromosomal *armA* genes were carried on genetic structures interrupting the histone acetyltransferase HPA2 gene (Fig. 4). In two of these isolates (A071 and A091), the genetic context of *armA* represented a composite transposon, labeled Tn*6279*, delineated by two peripheral copies of IS*26* (Fig. 4A). Tn*6279* was 20,959 bp long and included two segments sharing an internal copy of IS*26*. The first segment, Tn*6020b-1*, was 2,706 bp long and carried only the *aphA1b* gene. The second segment, Tn*1548*-like-1, was 20,862 bp long and carried six antimicrobial resistance genes [*aacA4*, *catB8*, *aadA1*, *armA*, *msr*(E), and *mph*(E)], with three of them (*aacA4*, *catB8*, and *aadA1*) being part of a class 1 integron. The Tn*1548*-like-1 segment also carried IS*CR1*, two transposase genes (*tnpU* and *tnpD*), one open reading frame (*orf*_Tn*1548*_) encoding a hypothetical protein, and a truncated plasmid replication initiator gene (Δ*rep*_Tn*1548*_). Tn*6279* was surrounded by a direct repeat of 8 bp (AGGATGAG). Isolate A070 carried a very similar structure, Tn*6279*-like, characterized by having a truncated copy of *aphA1b* on the first segment, Tn*6020b-2* (Table 5). Tn*6279*-like was also associated with an upstream deletion of 29,151 bp in the chromosome of A070 (Fig. 4A).

**TABLE 5:** Genetic structure and location of the *armA* elements

*armA* element	Genetic structure	Strain(s)	Location^*b*^	TSD^*a*^	Comment
Tn*6279*	IS*26*, ΔIS*26*, *aphA1b*, IS*26*, Δ*intI1*, *aacA4*, *catB8*, *aadA1*, *qacE*Δ1, *sul1*, IS*CR1*, *tnpU*, *armA*, *tnpD*, *msr*(E), *mph*(E), *orf*_Tn_*_1548_*-IS*Aba24*, Δ*rep*_Tn_*_1548_*, IS*26*	A071, A091	Chromosome	Yes	
Tn*6279*-like	IS*26*, ΔIS*26*, Δ*aphA1b*, IS*26*, Δ*intI1*, *aacA4*, *catB8*, *aadA1*, *qacE*Δ1, *sul1*, IS*CR1*, *tnpU*, *armA*, *tnpD*, *msr*(E), *mph*(E), *orf*_Tn_*_1548_*-IS*Aba24*, Δ*rep*_Tn_*_1548_*, IS*26*	A070	Chromosome	ND	Upstream deletion of 29,151 bp in the host chromosome
ΔTn*6279*	Δ*tnpU*, *armA*, *tnpD*, *msr*(E), *mph*(E), *orf*_Tn_*_1548_*-IS*Aba24*, Δ*rep*_Tn_*_1548_*, IS*26*	A072, A079, A080, A086, A089	Chromosome	ND	Upstream deletion of 3,937 bp in the host chromosome and 7,649 bp in the inserted element itself
ΔTn*6279*/IS*26*	Δ*tnpU*, *armA*, *tnpD*-IS*26*, *msr*(E), *mph*(E), *orf*_Tn_*_1548_*-IS*Aba24*, Δ*rep*_Tn_*_1548_*, IS*26*	A095	Chromosome	ND	Similar to ΔTn*6279* but with an additional copy of IS*26* interrupting the *tnpD* gene
Tn*1548*-like-2	Δ*intI1*, *arr-2*, *cmlA7*, Δ*qacE1*, *sul1*, IS*CR1*, *bla*_PER-7_, *gst*, Δ*abct*, ΔΔ*qacE1*, *sul1*, IS*CR1*, *tnpU*, IS*1999*, *armA*, *tnpD*, *msr*(E), *mph*(E), *orf*_Tn_*_1548_*, Δ*rep*_Tn_*_1548_*, IS*26*	A068, A092, A093, A096, A097	Plasmid	NA	Related to the Tn*1548*-like-1 segment of Tn*6279*
Tn*1548*-like-2-Δ1	Δ*gst*, Δ*abct*, ΔΔ*qacE1*, *sul1*, IS*CR1*, *tnpU*, IS*1999*, *armA*, *tnpD*, *msr*(E), *mph*(E), *orf*_Tn_*_1548_*, Δ*rep*_Tn_*_1548_*, IS*26*	A069	Plasmid	NA	Representing a truncated form of Tn*1548*-like-2; Δ*gst* was 237 bp
Tn*1548*-like-2-Δ2	Δ*gst*, Δ*abct*, ΔΔ*qacE1*, *sul1*, IS*CR1*, *tnpU*, IS*1999*, *armA*, *tnpD*, *msr*(E), *mph*(E), *orf*_Tn_*_1548_*, Δ*rep*_Tn_*_1548_*, IS*26*	A094	Plasmid	NA	Representing a truncated form of Tn*1548*-like-2; Δ*gst* was 314 bp

a TSD, target site duplication; ND, not detectable due to the upstream deletion; NA, not applicable.

b All the chromosomal *armA*-positive elements were inserted in *orf*_HPA2_, a histone acetyltransferase gene.

PCR assays using outwards internal primers, which were followed by sequence analysis of the PCR products, demonstrated the co-occurrence of circular forms of the Tn*1548*-like-1 and Tn*6020b* segments of Tn*6279* as well as a circular form of the entire Tn*6279* transposon, where only one copy of IS*26* was detected on each circular form (Fig. 4B). Furthermore, PCR assays using external primers on the sides of the insertion site showed that Tn*6279* was completely not present or was replaced by only one copy of IS*26* in parts of the population of each isolate. Concurrently, the occurrence of Tn*1548*-like-1, unaccompanied by Tn*6020b*, was also detected.

On the other hand, a truncated version of the Tn*6279* transposon, ΔTn*6279*, was detected in the other six isolates carrying a chromosomal *armA* gene (Table 5). ΔTn*6279* was formed by an upstream deletion of 11,586 bp, removing 3,937 bp of the host chromosome and 7,649 bp of the Tn*6279* transposon itself (Fig. 4A). The *tniD* gene was interrupted by an extra copy of IS*26* in isolate A095. Interestingly, *orf*_Tn*1548*_ was interrupted by IS*Aba24* in all the isolates with a chromosomally located *armA* gene regardless of whether the transposon was complete or partial.

The seven plasmid-mediated *armA* genes were carried on configurations showing a considerable structural similarity to the Tn*1548*-like-1 segment of Tn*6279* (Table 5). Five of these isolates had their *armA* carried on a configuration of 23,037 bp, labeled Tn*1548*-like-2, in which the *aacA4*, *catB8*, and *aadA1* gene cassettes of Tn*6279* were replaced by *arr-2* and *cmlA7*. In addition, Tn*1548*-like-2 was characterized by the occurrence of an extra segment carrying *bla*_PER-7_ and a second copy of IS*CR1*. Internal deletions of 7,984 and 7,907 bp were detected in the other two isolates (A069 and A094, respectively), creating the Tn*1548*-like-2-Δ1 and -Δ2 configurations, respectively. Both the complete and truncated forms of Tn*1548*-like-2 had an IS*1999* element inserted between *tnpU* and the *armA* gene. All these structures (namely, Tn*1548*-like-2, Tn*1548*-like-2-Δ1, and Tn*1548*-like-2-Δ2) were carried on large plasmids where they were surrounded by a configuration of 11,269 bp carrying a DNA methylase gene, few open reading frames encoding hypothetical proteins, *parA*, *parB*, *sul2*, and the IS*CR2* element on the right side and a configuration of 3,477 bp carrying a resolvase gene and a second IS*CR2* element on the left side. Interestingly, these *armA*-positive structures also coexisted as self-standing circular forms disconnected from the host plasmid (Fig. 4C).

The *aacC1*, *aacA4*, and *aadA1* genes were always carried on class 1 integrons, making four different arrays of gene cassettes (Table 4). The first array (*aacA4*, *catB8*, *aadA1*) was detected in three isolates and was located near the *armA* gene, as mentioned above. The second array (*aacC1*, *orfP*, *orfQ*, *aadA1*) was present in four isolates, where it was located in an AbaR3-like island (data not shown). The third array (*aacC1*, *orfP*, *orfP*, *orfQ*, *aadA1*) was present in an AbaR3- or AbGR12-like island in four isolates. A partial version of this array, due to an IS*26*-mediated deletion, was detected in the AbGR12-like island in 2 additional isolates (A077 and A087). The fourth array (*aacA4*, *aacC1*, *orfP*, *orfQ*, *aadA1*) was carried by only one isolate (A100), where it was located in an AbaR3-like island.

**FIG 5:**
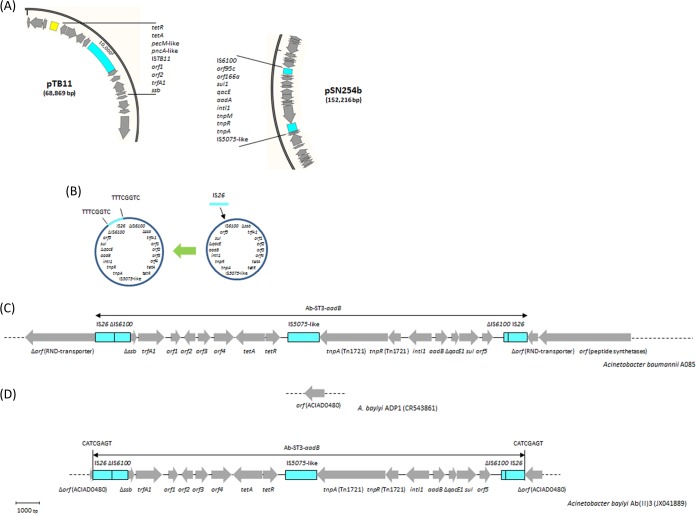
Potential sources, intermediate steps of formation, genetic structure, and site of acquisition of the Ab-ST3-*aadB* element. (A) Partial diagrams of plasmids pTB11 (GB accession number AJ744860) and pSN254b (GB accession number KJ909290), representing potential sources for the two segments of a prospective Ab-ST3-*aadB* element. Black lines, the segments involved in the construction of Ab-ST3-*aadB*. The coding regions of these segments were labeled according to GenBank records. (B) Circular intermediate forms of Ab-ST3-*aadB*. The acquisition of IS*26* was associated with a target site duplication, indicated by vertical lines. (C) Comparative analysis between Ab-ST3-*aadB*-negative Acinetobacter baumannii strain TCDC-AB0715 (GB accession number CP002522) and Ab-ST3-*aadB*-positive A. baumannii strains A085 (this study) and AB4857 (GB accession number AHAG01000030). The acquisition of Ab-ST3-*aadB* in A085 and AB4857 was not associated with a target site duplication. (D) Comparative sequence analysis between Ab-ST3-*aadB*-negative Acinetobacter baylyi strain ADP1 (GB accession number CR543861) and Ab-ST3-*aadB*-positive A. baylyi transformant Ab(II)3 (GB accession number JX041889). A. baylyi ADP1 was used as a recipient to detect the natural transformation of Ab-ST3-*aadB* from A. baumannii A064. The acquisition of Ab-ST3-*aadB* in A. baylyi Ab(II)3 was associated with a target site duplication, indicated by vertical lines. Arrows, coding regions oriented according to the direction of transcription.

The *aadB* gene in isolate A085 was also carried on a class 1 integron, which was located on a distinctive genetic structure of 18,625 bp named Ab-ST3-*aadB* (Fig. 5). In contrast, the *aadB* gene in isolate A078 was not located on an integron, although it was part of a gene cassette. Instead, this *aadB* gene was carried on a plasmid of 6,078 bp showing 100% nucleotide sequence identity to pRAY*-v1 (GB accession number JF343536). The *aacC2* gene, detected in seven isolates, was not carried on class 1 integrons (Table 4). In six isolates, *aacC2* was surrounded by IS elements in a structure showing 100% nucleotide sequence identity to a corresponding sequence in A. baumannii AB5256 (GB accession number AHAI01000050). Noticeably, these six *aacC2*-positive isolates and strain AB5256 belonged to ST25 (data retrieved from the GB sequence). The last *aacC2* gene, in isolate A082, was surrounded by different IS elements, where it was located next to a gene encoding an ABC-ATPase protein and two open reading frames encoding hypothetical proteins.

**FIG 6:**
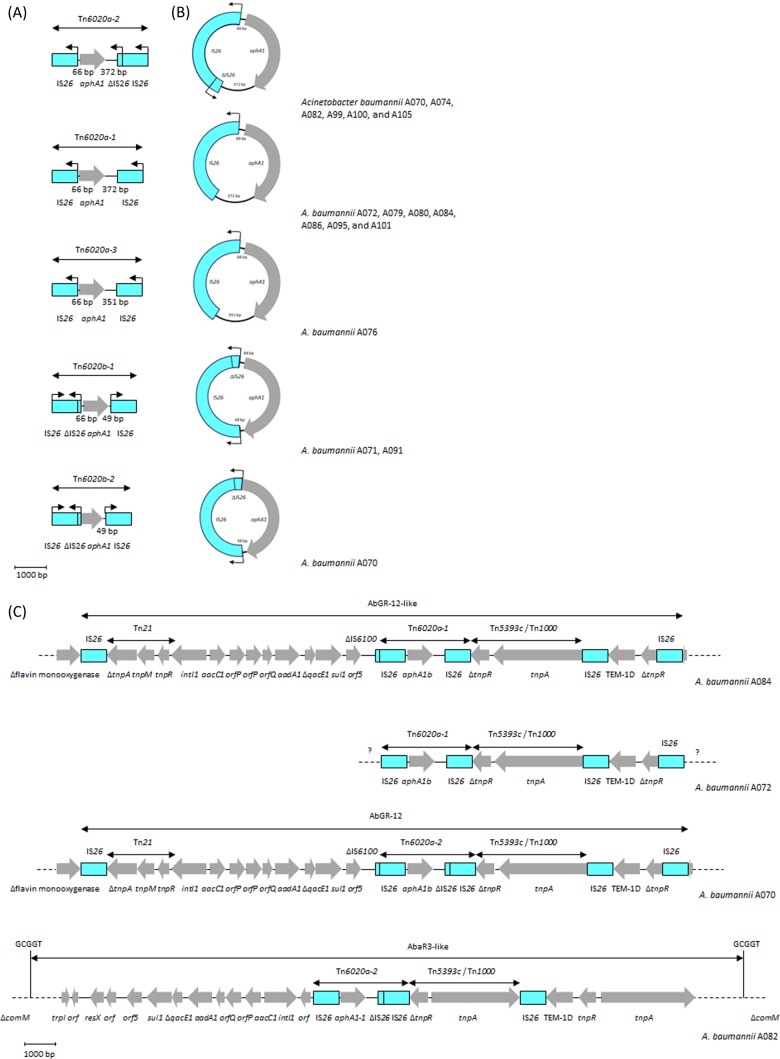
Genetic context of *aphA1*. (A) Genetic structures of Tn*6020a-1*, Tn*6020a-2*, Tn*6020a-3*, Tn*6020b-1*, and Tn*6020b-2*. (B) Co-occurrence of transposon-derived circular forms corresponding to Tn*6020a-1*, Tn*6020a-2*, Tn*6020a-3*, Tn*6020b-1*, and Tn*6020b-2*. (C) Genetic context and location of Tn*6020a-1* or Tn*6020a-2* in isolates A084, A072, A070, and A082. Gray arrows, genes, as labeled, with the arrowhead indicating the direction of transcription; blue boxes, insertion sequence elements. In panels A and B, the labeled blue boxes were decorated with black arrows indicating the direction of transcription of the IS*26* transposase gene; the lengths of the spacers upstream and downstream of *aphA1b* are presented in base pairs, and the structures were drawn to the indicated scale.

The *aphA1* gene was surrounded by IS*26* elements in all our positive isolates (Fig. 6). Five different assemblies were defined in our isolates, including two assemblies carried by one isolate (A070) (Fig. 6A). The first assembly, Tn*6020a-1*, was present in seven isolates that were all from CC2 (Table 4). In this assembly, *aphA1b* was transcribed in the opposite direction compared to the surrounding IS*26* elements, and the spacers between *aphA1b* and the upstream and downstream IS*26* elements were 66 and 372 bp, respectively. Tn*6020a-1* showed a nucleotide sequence identity of 99 to 100% to a few isolates in the GB databases, such as Proteus mirabilis strain PmCHA and A. baumannii strain ab929679598 (GB accession numbers KJ411925 and JMOT01000069, respectively).

The second assembly, detected in six isolates from different CCs, was almost identical to Tn*6020a-1*, except that the downstream IS*26* was truncated by another copy of IS*26*. This assembly, designated here as Tn*6020a-2* only for the use of this article, showed 100% structural and nucleotide sequence identity to Tn*6020* (representing the endorsed designation of this structure), which has been described among several A. baumannii isolates from Australia (9). The third assembly, Tn*6020a-3*, was also related to Tn*6020a-1*. It was characterized by a downstream spacer of only 351 bp and was present in one isolate (A076). In contrast to Tn*6020a-1*, -*2*, and -*3*, the *aphA1b* gene in Tn*6020b-1* and -*2* was transcribed in the same direction as the two intact peripheral IS*26* elements. The upstream IS*26* was truncated by another copy of IS*26*, and the spacer between *aphA1b* and the downstream IS*26* element was only 49 bp. Tn*6020b-1*, detected in two isolates, was found to be plasmid mediated in Escherichia coli pMUR050 (GB accession number AY522431) and A. baumannii/p2BJAB07104, A. baumannii/p3BJAB0868, and A. baumannii/pMDR-ZJ06 (GB accession numbers CP003907, CP003908, and CP001938, respectively). Lastly, Tn*6020b-2* was carried by only one isolate (A070) and was identical to Tn*6020b-1*, except that it lacked the upstream spacer and the first 94 bp of *aphA1b*.

**FIG 7:**
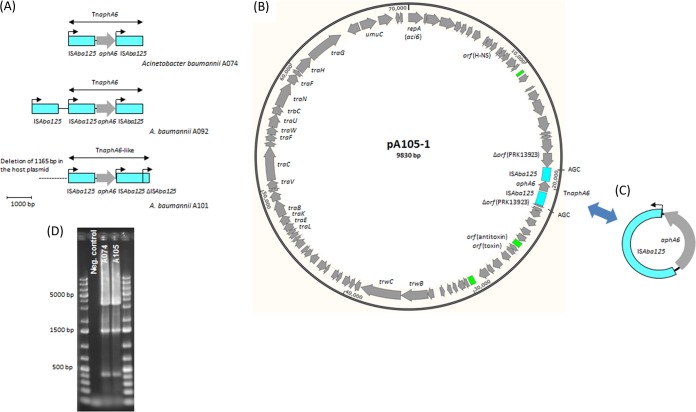
Genetic context of *aphA6*. (A) Genetic structure of Tn*aphA6* and Tn*aphA6*-like. Gray arrows, coding regions, with the arrowhead indicating the direction of transcription; blue boxes, IS*Aba125* elements; black arrows, the direction of transcription of the transposase gene. Tn*aph6* is highlighted. The structures were drawn to the indicated scale. (B) Genetic structure of plasmid pA105-1 (this study). Gray arrows, coding regions, with the arrowhead indicating the direction of transcription; blue and green boxes, IS*Aba125* and regions of repeated sequences, respectively. (C) Genetic structure of the circular form of Tn*aphA6*. (D) Gel electrophoresis of PCR products amplified using primers surrounding the insertion site of Tn*aphA6* in isolates A074 and A105. The detection of three bands indicated that the population of each isolate could be divided into three parts, namely, part 1, where the site was intact (small band of 423 bp); part 2, where the site was inserted only by IS*Aba125* (medium-sized band of 1,513 bp); and part 3, where the site was inserted by Tn*aphA6* (large band of 3,498 bp).

Likewise, *aphA6* was always surrounded by IS*Aba125* elements (Fig. 7). In 10 isolates, *aphA6* was surrounded by two copies of IS*Aba125*, creating a Tn*aphA6a* transposon, similar to what has previously been found on a variety of A. baumannii plasmids, such as pWH8144, pBJAB0715, pAb-G7-2, pD72-2, and pD46-3 (GB accession numbers NG_041543, CP003848, KF669606, KM051846, and KM977710, respectively). In one isolate (A101), the downstream IS*Aba125* was truncated by another IS*Aba125*, producing the Tn*aphA6*-like transposon with a genetic structure that has not been reported before (Fig. 7A).

## DISCUSSION

### International clones and limited outbreaks.

Along with the extensive worldwide dissemination of CC2 (2), nearly half of our isolates belonged to this clone. Fine molecular typing demonstrated that four of the CC2 isolates (A072, A079, A080, and A086) represented an outbreak of infections taking place in one county of Sweden between September 2012 and April 2013 (Table 1; see also Fig. S2 in the supplemental material). As in previous studies, an epidemiological linkage between the CC2 clone and the *bla*_OXA-66_ and *bla*_ADC-30_ variants was detected (33, 34). Interestingly, seven of the CC2 isolates carried *bla*_ADC-73_, a novel variant of *bla*_ADC_ with a sequence identity of 1,151/1,152 nucleotides compared to the sequence of *bla*_ADC-30_. *bla*_ADC-73_ has previously been detected in the genome of only a few A. baumannii isolates, such as AC12 and PKAB07 (35, 36). Of note, both AC12 and PKAB07 also belong to CC2 (data retrieved from the GB sequences).

The existence of seven CC25 isolates in our collection was probably biased by the occurrence of another small outbreak of infections that took place in one county in August 2013. SNP-based phylogenetic analysis and PFGE also confirmed the occurrence of this outbreak (see Fig. S2 in the supplemental material). The findings of our study are consistent with a growing number of reports describing CC25 to be a highly successful clone with an extensive international spread (2, 37, 38). As described elsewhere, the *bla*_OXA-64_ and *bla*_ADC-26_ variants have generally been characteristic for the CC25 clone (34, 38).

Although the occurrence of international clone CC1 has generally been decreasing (2), three of our isolates belonged to this clone. However, the three CC1 isolates carried different variants of the *bla*_ADC_ gene and belonged to different branches according to the SNP-based phylogenetic analysis and PFGE results. Similarly, the epidemiology of the two ST636 isolates indicated the occurrence of independent acquisitions. Interestingly, the ST215 isolate carried both *bla*_OXA-66_ and *bla*_ADC-30_, indicating that it could be a remote subclone of CC2. One isolate belonged to ST23/CC10, a clone that has so far been restricted to Europe and Australia (2). Two Swedish isolates from our previous study in 2011 also belonged to CC10 (14). Nonetheless, an epidemiological connection between the isolates could not be proposed due to the long gap in the time of acquisition.

According to our knowledge, no formalized numbering scheme has been published for the *bla*_ADC_ gene. The gene is also not included in the Lahey Clinic databases of β-lactamases (http://www.lahey.org/Studies/). Accordingly, we made a table of the currently available ADC variants that have been fully sequenced (see Table S6 in the supplemental material). Since *bla*_ADC-72_ was the latest variant present in GenBank, our novel variants were assigned the numbers 73 to 81. Two *bla*_ADC_ variants with different amino acid sequences were concomitantly designated *bla*_ADC-57_ (GB accession numbers JQ037817 and HQ258925). In order to avoid duplications, *bla*_ADC-57_ was assigned to the variant that was detected in two A. baumannii isolates from East Africa (37). It is important to state that since *bla*_ADC_ is an *ampC* gene intrinsic in all the Acinetobacter spp., a new designation (such as *ampC*_Ac_) is probably required for precise terminology.

### Resistance to cefotaxime and ceftazidime.

In line with the findings of previous studies, IS*Aba1-bla*_ADC_ was the main mechanism of resistance to cefotaxime and ceftazidime in our isolates (33, 39). Of note, one of the isolates (A091) carried a second copy of IS*Aba1* located ∼2,000 bp downstream of the *bla*_ADC_ gene. The insertion of the two copies of IS*Aba1* most likely took place independently since each IS*Aba1* had its own 9-bp target site duplication. However, the two IS*Aba1* copies constructed a genetic structure that could act as a potential composite transposon in the same way that the transposon recently identified by Hamidian and Hall in a few A. baumannii isolates from Australia does (39). The IS*Aba125-bla*_ADC-81_ element showed 100% nucleotide sequence identity to a sequence present in A. baumannii strain A388, obtained in Greece, including the GCCCCTGCATATGGC internal duplication in *bla*_ADC-81_ (GB accession number JQ684178) (6). The DNA sequences flanking the acquired *bla*_ADC-77_ gene, present in isolate A078, included an upstream IS*6*-family element, similar to the genetic structure that has previously been described in Oligella urethralis (40). However, the occurrence of a downstream IS*1009* interrupted by another IS*6*-family element was present only in our isolate and not in the O. urethralis strain. The nucleotide sequence identity of the internal segment shared by the two structures was 99.8%. A possible origin for this internal segment was found in the draft genome of A. baumannii isolate NIPH 1734 (GB accession number APOX01000011), in which *bla*_ADC_ was most likely intrinsic (data not shown).

Six of the cefotaxime- and ceftazidime-resistant isolates carried *bla*_PER-7_, and interestingly, all of them belonged to ST25. The increased activity of *bla*_PER-7_ against broad-spectrum cephalosporins has been reported in an A. baumannii isolate from France and an ST25 isolate from the United Arab Emirates (UAE) (38, 41). The French isolate probably also belonged to ST25 since it carried the characteristic *bla*_OXA-64_ gene, as mentioned in a previous paragraph. As in the UAE strain, the spontaneous loss of the *bla*_PER-7_ gene and the nearby integron took place in one of the five isolates representing the Östergötland ST25 outbreak strain, leaving this isolate susceptible to ceftazidime (41).

### Acquisition and construction of Tn*6279*.

Only one copy of IS*26* was inserted in the *orf*_HPA2_ gene in the genomes of A. baumannii strains BJAB07104 and BJAB0868 (GB accession numbers CP003846 and CP003849, respectively). The occurrence of a target site duplication of 8 bp indicated that the acquisition of this IS*26* copy, representing the first step in the acquisition of Tn*6279*, was mediated by a typical transposition event. The second step involved the acquisition of Tn*1548*-like-1, as demonstrated by our results and in A. baumannii strains TYTH-1 and MDR-TJ (GB accession numbers CP003856 and CP003500, respectively), in which the *orf*_HPA2_ gene was interrupted only by Tn*1548*-like-1. The acquisition of Tn*1548*-like-1 was most likely mediated by a homologous recombination event between the IS*26* copy that was already present in this location and a second copy of IS*26* carried on a circular form of Tn*1548*-like-1 (42). This segment was labeled on the basis of the similarity of its genetic structure to that of Tn*1548* (GB accession number JN225877), a transposon that has repeatedly been found in the Enterobacteriaceae family (43, 44). The only difference between Tn*1548* and our derivative, Tn*1548*-like-1, was in the gene cassettes of their class 1 integrons. Since the two segments of Tn*6279* shared only one in-between IS*26* copy, the acquisition of Tn*6020b-1*, representing the third and last step in the acquisition of Tn*6279*, was also mediated by a homologous recombination event taking place between the left-sided IS*26* copy of Tn*1548*-like-1 and a copy of IS*26* carried on an intermediate circular form of Tn*6020b-1* (45).

A previous study has reported the chromosomal occurrence of Tn*6279* in A. baumannii strain NCGM 237 (GB accession number AP013357), representing 49 A. baumannii isolates collected by different hospitals in Japan (33). However, Tn*6279* in NCGM 237 was present at a different location and was not surrounded by an 8-bp duplication, most likely because the acquisition took place using a chromosomal IS*26* element inserted from the beginning without creating a target site duplication. A 20-kb Tn*6279*-derived plasmid was detected in strains BJAB07104 and BJAB0868 (GB accession numbers CP003907 and CP003908, respectively) (46). A similar plasmid equipped with a different array of gene cassettes was present in A. baumannii strain MDR-ZJ06 (GB accession number CP001938). A structure related to Tn*6279* was also found to be inserted in a large plasmid of 57 kb held by an Escherichia coli isolate (GB accession number AY522431) (44). On the other hand, ΔTn*6279* was found in the genome of A. baumannii strain PKAB07 (GB accession number CP006963), reported to be a carbapenem-resistant isolate recovered from India in 2011-2012 (36). Of note, all these *armA*-positive A. baumannii strains, namely, TYTH-1, MDR-TJ, BJAB07104, BJAB0868, NCGM 237, MDR-ZJ06, and PKAB07, belonged to CC2 and were isolated from East Asia (16, 46–48; data retrieved from the GB sequences).

Our *armA*-positive plasmids showed a close structural similarity to pIOMTU433 (GB accession number AP014650), recovered from an A. baumannii isolate obtained in Japan, and pAB04-1 (GB accession number CP012007), carried by an A. baumannii isolate responsible for an outbreak of infections in Canada, although it had a history of import from India (49). In addition to the geographic dispersal, the last two isolates did not belong to CC25 (49; data retrieved from the GB sequences). The Δ*rep*_Tn*1548*_ replication gene of the Tn*1548*-like elements and their corresponding circular forms shared nucleotide sequence similarities of 85 to 90% with genes encoding the plasmid replication proteins Aci7 (GB accession number NG_040962) and Aci3 (GB accession number NG_040963). Accordingly, Δ*rep*_Tn*1548*_ should probably be classified into group 3 using the AB-PBRT system (25).

### Different genetic environments for *aadB*.

On the basis of their structural similarities, the *aadB*-positive pRAY*-v1 plasmid was reported to be a derivative of pRAY, which was first detected in an Acinetobacter species isolate recovered in South Africa (50). Later on, different variants of pRAY, including pRAY*-v1, were detected among several Acinetobacter species isolates from Australia (8, 51, 52). A similar plasmid was also present in one A. baumannii strain isolated in the Netherlands in 1984 (52). These plasmids were equipped with the *mobA* and *mobC* mobilization genes, but there was no gene encoding a potential replication initiation protein. The plasmids also carried a gene encoding the Abi-like protein (pfam07751) involved in bacteriophage resistance mediated by abortive infection in Lactococcus species (53).

The Ab-ST3-*aadB* structure carried a segment that had probably been obtained from one of the IncP1α plasmids, such as pTB11 (GB accession number AJ744860), without the IS*TB11* element, which were detected in uncultured bacteria or Pseudomonas aeruginosa (Fig. 5A) (54, 55). Several genes were identified on this segment, including the *ssb-trfA* operon, involved in initiation of plasmid replication, and the *tetA* tetracycline resistance gene (55). It is important to mention that the nucleotide sequence of the *tetA* gene in Ab-ST3-*aadB* (isolate A085) had 8 point mutations compared to the sequences of the *tetA* genes of our other isolates, such as A082 and A099 (see Fig. S4 in the supplemental material). In contrast to the structures of the IncP1α plasmids, the *ssb* gene was truncated by IS*6100*, and many other genes, including the origin of vegetative replication (*oriV*), were missing in our structure (55).

The other main segment of Ab-ST3-*aadB* was derived from a different group of plasmids, such as pSN254b (GB accession number KJ909290), detected in a fish-pathogenic strain of Aeromonas salmonicida subsp. salmonicida (56). However, a different class 1 integron gene cassette was detected on plasmid pSN254b. This segment included IS*5075*-like, Tn*1721*-like (*tnpA-tnpR*), and class 1 integron (*intI1-aadB*-Δ*qacE-sul1-orf5*) regions. Interestingly, this segment ended with an IS*6100* element, most likely representing the same copy of IS*6100* that truncated *ssb* of the first segment. IS*6100* was then interrupted by an IS*26* element, associated with the 8-bp target site duplication (Fig. 5B). Similar to the findings for Tn*6279*, PCR assays demonstrated the occurrence of an intermediate circular form of Ab-ST3-*aadB* and showed that part of the population of this isolate carried an IS*26* element only in the insertion site.

Ab-ST3-*aadB* showed 100% nucleotide sequence identity to sequences from A. baumannii strain AB4857 (GB accession number AHAG01000030). Nonetheless, AB4857 had a deletion of 8,067 bp in the adjacent downstream region compared to the sequence of our isolate (Fig. 5C). The absence of surrounding target site duplications both in A085 and in AB4857 indicated that the acquisition of Ab-ST3-*aadB* was due to an IS*26*-mediated homologous recombination event involving the circular form of Ab-ST3-*aadB* and an IS*26* copy already present in the chromosome (42). During the acquisition process, the inserted Ab-ST3-*aadB* became flanked by two copies of IS*26*, splitting the IS*6100* element into two parts (Fig. 5C and D). Ab-ST3-*aadB* has broadly and exclusively been present in A. baumannii isolates belonging to ST3. The isolate carrying Ab-ST3-*aadB* in our collection also belonged to ST3, proposing that this element represents a genetic marker with a potential use in tracking the occurrence of ST3 isolates. A previous study reported on the natural transformation of Ab-ST3-*aadB* from A. baumannii 064 into the genome of Acinetobacter baylyi ADP1 (GB accession number CR543861), resulting in transformant A. baylyi Ab(II)3 (GB accession number JX041889) (57). In the A. baylyi recipient, Ab-ST3-*aadB* interrupted a gene (labeled ACIAD0480, according to GB records), where the acquisition created a target site duplication of 8 bp (Fig. 5D). Accordingly, the acquisition of Ab-ST3-*aadB* by A. baylyi Ab(II)3 was due to natural transformation followed by standard transposition of the entire IS*26* composite transposon (57).

### Characterization of the Tn*6020* and Tn*aphA6* transposons.

All the *aphA1*-positive isolates in our collection carried *aphA1b*, a variant that was first detected in a Proteus rettgeri strain more than 40 years ago (58). Previous studies have reported the occurrence of *aphA1b* surrounded by at least two complete copies of IS*26* (9, 27). The genetic context of Tn*6020a-1* in isolate A084 (CC2) and Tn*6020a-2* in isolate A070 (CC215) corresponded to the AbGR-12 island (Fig. 6B). The occurrence of Tn*6020a-2* in AbGR-12 was previously described in A. baumannii strains WM99c and A94, from the CC2 collection of A. baumannii isolates obtained in Australia (27). However, the replacement of Tn*6020a-2* by Tn*6020a-1* in this location was novel and highlighted the plasticity of the AbGR-12-like islands. On the other hand, Tn*6020a-2* in isolates A082 (CC1), A99 (ST85), and A100 (CC1) and Tn*6020-3* in isolate A076 (CC1) were all located in an AbaR3-like background, similar to what has previously described in a number of CC1 A. baumannii strains, such as RUH875, WM98, AB0057, and AYE (9). In addition to *aphA1*, the AbGR-12- and AbaR3-like islands have frequently been equipped with *bla*_TEM-D1_ and a class 1 integron(s) (48). Tn*6020a-1* was also linked to *bla*_TEM-D1_ in the other six positive isolates (along with isolate A084). However, the whole construct was not carried in either an AbaR3- or an AbGR12-like island in these isolates. Noticeably, none these six isolates carried class 1 integrons (Table 4).

As described above and in previous studies, Tn*6020b-1* and Tn*6020b-2* were associated with the *armA* gene (16, 46). Circular forms of Tn*6020* were detected in most of the *aphA1*-positive isolates (Fig. 6C). The size of each circular form was consistent with the length of the corresponding assembly (see Fig. S5 in the supplemental material). Tn*6020* was consistently displaced in a fraction of the population of each of these isolates. Our results are in line with those of a number of previous studies reporting on the readiness of the IS*26*-flanked composite transposons to be excised out, leading to the formation of circular intermediate forms (59, 60). A recent study demonstrated that the acquisition of IS*26*-based circular forms was RecA independent (42). Instead, it was reliant on the presence of an intact *tnp26* transposase gene. According to Harmer et al. (42), the frequency of being integrated was 60-fold higher when the target site contained a parallel IS*26* element.

The *aphA6* kanamycin and amikacin resistance gene was first detected in A. baumannii strain BM2580, isolated in 1984, in which the gene was carried on a 63-kb self-transferable plasmid (61). Structural comparisons of the previously detected *aphA6*-positive plasmids demonstrated a distinctive construction for pWH8144 and pBJAB0715, both of which are equipped with Tn*aphA6* and *bla*_OXA-58_ (46, 62). On the other hand, the pAb-G7-2, pD72-2, and pD46-3 plasmids had a common *aci6*-positive backbone, although they were detected in isolates belonging to three different CCs, namely, CC1, CC2, and CC25 (8, 63).

Tn*aphA6* in six of our isolates was inserted at the same location at which it was located in pAb-G7-2 (64). A third copy of IS*Ab125* was present close to Tn*aphA6* in the ST25 isolates (Fig. 7A). Tn*aphA6*-like was also inserted at this location, but it was associated with an upstream deletion of 1,165 bp in the host plasmid. In the two ST636 isolates, Tn*aphA6* was carried on a plasmid of ∼70 kb showing a considerable structural similarity to pAb-G7-2, pD72-2, and pD46-3, although it was inserted at a new site and in the opposite direction compared to its location and direction in pAb-G7-2 (Fig. 7B). This plasmid, labeled pA105-1, carried a type IV secretion system and at least one toxin-antitoxin system, as previously described for the *aci6* plasmids (63). In the remaining two isolates, Tn*aphA6* was most likely located on a novel carrier, which we were not able to define. As expected, these last two isolates were deprived of *aci6* plasmids (see Table S4 in the supplemental material). All the *aphA6*-positive isolates carried coexisting circular forms of Tn*aphA6* (Fig. 7C), indicating that IS*Aba125* (from the IS*30* family) had the same ability as IS*26* (from the IS*6* family) to create enduring intermediate circular forms. All the IS*Aba125*- and IS*26*-based circular forms that were detected in our study had only one copy of the corresponding IS element. The site of insertion of Tn*aphA6* in the two ST636 isolates was intact (in one part of the population of each isolate), inserted by only one IS*Aba125* (in a second part), or inserted by Tn*aphA6* (in a third part), as illustrated in Fig. 7D.

Overall, our study precisely defined the genetic context of particular aminoglycoside resistance genes in A. baumannii, leading to the identification of a few novel genetic structures, such as Tn*6279* and Ab-ST3-*aadB*, and a number of circular forms related to the IS*26* or IS*Aba125* composite transposons. The potential role of these circular forms in the dissemination of antibiotic resistance genes needs further investigation.

## Supplementary Material

Supplemental material
