# Supplementary material for: Novel Aminoglycoside Resistance Transposons and Transposon-Derived Circular Forms Detected in Carbapenem-Resistant Acinetobacter baumannii Clinical Isolates
Source: Antimicrob Agents Chemother. 2016 Feb 26;60(3):1801–18. doi: 10.1128/AAC.02143-15 (PMC4776018; doi:10.1128/AAC.02143-15)
Supplement: Supplemental material [file supp_60_3_1801__index.html]

Novel Aminoglycoside Resistance Transposons and Transposon-Derived Circular Forms Detected in Carbapenem-Resistant Acinetobacter baumannii Clinical Isolates — Supplemental material 

# Novel Aminoglycoside Resistance Transposons and Transposon-Derived Circular Forms Detected in Carbapenem-Resistant Acinetobacter baumannii Clinical Isolates

## Supplemental material

- Supplemental file 1 -

  Tables S1-S6 and Fig. S1-S5

  PDF, 1.4M
