# Supplementary material for: Novel Aminoglycoside Resistance Transposons and Transposon-Derived Circular Forms Detected in Carbapenem-Resistant Acinetobacter baumannii Clinical Isolates
Source: Antimicrob Agents Chemother. 2016 Feb 26;60(3):1801–18. doi: 10.1128/AAC.02143-15 (PMC4776018; doi:10.1128/AAC.02143-15)
Supplement: Supplemental material [file AAC.02143-15_zac003164959so1.pdf]

SUPPLEMENTAL MATERIAL

TABLE S1. Primers and PCR assays.

PCR assay title	PCR assay code (genetic target)	Oligo Name	Oligo features	5'-3' sequence	Expected amplicon size(bp)
Genetic context of *bla*$_{ADC}$	PCR1 (ADC upstream)	folE/ADC-F	External, inwards	GCTGAACGCGATAAACTTCG	814 / 1902 / 2003
		ADC-R	Internal, outwards	TCGAATCGGCTGATTTTCTT	
	PCR2 (ADC downstream)	ADC-F	Internal, outwards	CTGGCTCAACTACCGGTTTC	2360 / 3551
		ADC/orf-R	External, inwards	GCTGTAGGCCAAGGTGAAAG	
Genetic context of *bla*$_{OXA-51-like}$	PCR3 (OXA-51-like upstream)	fxsA-F	External, inwards	TTTGCGAGTGTTCTCATTGC	1706 / 2895
		N-Acyltransferase-R	External, inwards	GGCTTGACGCTGCTTTTTAC	
Genetic context of *bla*$_{OXA-23}$	PCR4 (Tn2006 downstream)	Tn6022-sup-F	External, inwards	CCTTGCCCTTTACATTCTCG	3309
		ΔDEAD-R	Internal, outwards	ATGGGCTTCGTCATTCATTG	
	PCR5 (Tn2006 upstream)	ΔDEAD-F	Internal, outwards	GTGCCGCCTATCAGTTCTCT	2688
		Tn6022-orf4-R	External, inwards	GAAAATGCCAAGGGAATAAAAA	
	PCR6 (Tn2009 downstream)	fimC-F	External, inwards	TTTTGTCGCTTCAACACGAG	1610
		Tn2009-orf-R	Internal, outwards	ATCGCCACGGAAGTATGAAC	
	PCR7 (Tn2009, OXA-23 downstream)	Tn2009-T5orf172-F		CTTTCTTTGGTGAGGCTTGC	2484
		ΔDEAD-R		ATGGGCTTCGTCATTCATTG	
	PCR8 (Tn2009 upstream)	ΔDEAD-F	Internal, outwards	GTGCCGCCTATCAGTTCTCT	2441
		fimA-R	External, inwards	TCAATGGGTTGACGTAGCTG	
Genetic context of *bla*$_{OXA-24-like}$	PCR9 (OXA-72-positive Aci1 plasmid)	pAB0057-orf-F		AATACGATCGGCTTGCTGTT	1609
		pAB0057-repB-R		GACGCGCGAATAAATCCTTA	
	PCR10 (OXA-24-positive Aci1 plasmid)	pABVA01-orf-F		ATACGATCTGCCTGCTGCTT	1732
		pABVA01-repB-R		CTATGGCTGGGGCAAAGATA	
	PCR11 (OXA-24-like downstream)	repA-F	External, inwards	TACCACACCCAAACGCTACA	635
		oxa-24-R	Internal, outwards	CAAAAAGTGGATGGGGAATG	
	PCR12 (OXA-24-like upstream)	oxa-24-F	Internal, outwards	GCTTTTTCATGTTGCTGAGAA	1438 / 2298
		tonB-R	External, inwards	CCTGCCCACGAATAATAGGA	
	PCR13 (OXA-72-positive Aci1 plasmid - isolate A100)	pA100-F-1		CGAGGGCGAAATTTTAATGA	445
		pA100-emrE-R		ACCATCACGCTTGGAAAAAG	
	PCR14 (OXA-72-positive Aci1 plasmid - isolate A100)	pA100-F-2		GCTTGCAAAGCAAGTGGATA	1011
		pA100-mobA-R		GTGGTTTAAGCCGTGTCGAT	
	PCR15 (OXA-72-positive Aci1 plasmid - isolate A100)	pA100-F-1		CGAGGGCGAAATTTTAATGA	3240
		pAB0057-repB-R		GACGCGCGAATAAATCCTTA	
	For additional sequencing	ISAba31-F		AACGAATAGCCAATCGAACAA	

TABLE S1. CONT. Primers and PCR assays.

PCR assay title	PCR assay code (genetic target)	Oligo Name	Oligo features	5'-3' sequence	Expected amplicon size(bp)
Genetic context of *bla*$_{OXA-58}$	PCR16 (OXA-58 downstream)	hsdS-F	External, inwards	CGTGGAGCTACATGTGGTGA	2428
		oxa-58-R	Internal, outwards	GGGTTGGTATGTGGGTTTTG	
	PCR17 (OXA-58 upstream)	oxa-58-F	Internal, outwards	AAAAGCGCTTGAACATTCTGA	2242
		cinH-R	External, inwards	GGATTTTTGTTGACCGTGCT	
	For additional sequencing	ISAba3-R		AGAGGCAGCGGTATACGAGA	
Genetic context of *aadB1*	PCR18 (Ab-ST3-*aadB1* upstream)	MMPL-F	External, inwards	TCTTGGCATCGAACCCTTCT	1332
		TnST3-IS6100-R	Internal, outwards	GGCTTTTCCCAGTGCTTCAG	
	PCR19 (Ab-ST3-*aadB1* downstream)	TnST3-IS6100-F	Internal, outwards	AGGGTGATGTGATCCTGTGG	1150
		MMPL-R	External, inwards	AGGACCCTTTCACTACACCG	
	PCR20 (Ab-ST3-*aadB1* circular form)	TnST3-IS6100-F	Internal, outwards	AGGGTGATGTGATCCTGTGG	1192
		TnST3-IS6100-R	Internal, outwards	GGCTTTTCCCAGTGCTTCAG	
	PCR21 (Ab-ST3-*aadB1* circular form)	TnST3-F	Internal, outwards	TCAGAAGACGACTGCACCAG	2008
		TnST3-ssb-R	Internal, outwards	ATCTTGCCCTGCACGAATAC	
	PCR22 (Ab-ST3-*aadB1* insertion site)	MMPL-F	External, inwards	TCTTGGCATCGAACCCTTCT	1290
		MMPL-R	External, inwards	AGGACCCTTTCACTACACCG	
	PCR23 (*aadB1*-positive plasmid pRAY*-v1)	mobA-F		GCTGTACTTCGTTCTAGCTCG	1228
		mobA-R		TGTGATTCCCAATGTTGAGC	
Genetic context of *armA*	PCR24 (Tn*6279* upstream, linkage between host chromosome and Tn*6020b*)	HPA2-F	External, inwards	GCTGCACAGTGGTTTTCTGA	1967
		aphA1b-F (R)	Internal, outwards	CGACTGAATCCGGTGAGAAT	
	PCR25 (Tn*6279* upstream, linkage between host chromosome and Tn*6020b*)	araC-F	External, inwards	AATGCCAATGGGGATCATAA	1801
		aphA1b-F (R)	Internal, outwards	CGACTGAATCCGGTGAGAAT	
	PCR26 (Tn*6279* upstream, linkage between host chromosome and Tn*1548*-like-1)	HPA2-F	External, inwards	GCTGCACAGTGGTTTTCTGA	3214
		catB8-R	Internal, outwards	GCACCGTCTCCAATTTTGAT	
	PCR27 (Tn*6279* upstream, linkage between host chromosome and Tn*1548*-like-1)	araC-F	External, inwards	AATGCCAATGGGGATCATAA	3208
		catB8-R	Internal, outwards	GCACCGTCTCCAATTTTGAT	
	PCR28 (ΔTn*6279* upstream, linkage between host chromosome and ΔTn*6279*)	sig-pep-F	External, inwards	AAAGTGACTCCGGTCTCGAA	507
		tnpU-R	Internal, outwards	GTGGCATCAATGACCAAGTG	
	PCR29 (Tn*6020b*)	IS26-F		TGGTACTGGCGTAACCCTTC	1180 / 1349
		aphA1b-F (R)		CGACTGAATCCGGTGAGAAT	
	PCR30 (Tn*6020b*)	aphA1b-R (F)		GGTTGCATTCGATTCCTGTT	931
		IS26-R1		TCTGCCACTTCTTCACGTTG	
	PCR31 (linkage between Tn*6020b* and Tn*1548*-like-1)	aphA1b-R (F)		GGTTGCATTCGATTCCTGTT	3325
		catB8-R		GCACCGTCTCCAATTTTGAT	
	PCR32 (Tn*1548*-like-1)	catB8-F		ATGACTGGGCATCATCCTTC	3114
		CR1-R		GCTTTGAAGGCTGGGTAGTG	

TABLE S1. CONT. Primers and PCR assays.

PCR assay title	PCR assay code (genetic target)	Oligo Name	Oligo features	5'-3' sequence	Expected amplicon size(bp)
Genetic context of *armA*	PCR33 (Tn*1548*-like-1)	catB8-F		ATGACTGGGCATCATCCTTC	886
		aadA1-R		ACCAAGGCAACGCTATGTTC	
	PCR34 (Tn*1548*-like-1)	aadA1-F		TGATTTGCTGGTTACGGTGA	2522
		CR1-R		GCTTTGAAGGCTGGGTAGTG	
	PCR35 (Tn*1548*-like-1)	aadA1-F		TGATTTGCTGGTTACGGTGA	4462
		tnpU-R		GTGGCATCAATGACCAAGTG	
	PCR36 (host plasmid, adjacent to Tn*1548*-like-2, -Δ1 and -Δ2)	CR2-F		ATCAAAATGCTGCGTTCACA	1044
		sulI2-R		GATATTCGCGGTTTTCCAGA	
	PCR37 (host plasmid, adjacent to Tn*1548*-like-2, -Δ1 and -Δ2)	CR2-F		ATCAAAATGCTGCGTTCACA	2338
		parA-R		ATGGGCTCGTAGTCACCATC	
	PCR38 (linkage between the host plasmid and Tn*1548*-like-2)	Tnsul2-F		GAATTGCCTTTTGGCGTTTA	3546
		arr2-R		GCTCCATCAAGGCTGAAAAG	
	PCR39 (linkage between the host plassmid and Tn*1548*-like-2-Δ1 and -Δ2)	Tnsul2-F		GAATTGCCTTTTGGCGTTTA	2543 / 2620
		gst-R		AATCGCGGAACAATATCCAG	
	PCR40 (Tn*1548*-like-2)	cmlA5-F		CTGCTAGGAAGCATCGGAAC	3889
		PER7-R		TCAGTAGCGTCGAGGCAGTA	
	PCR41 (Tn*1548*-like-2)	PER7-F		AGCTATCATTGCGCAGGTTG	630
		gst-R		AATCGCGGAACAATATCCAG	
	PCR42 (Tn*1548*-like-2, -Δ1, and -Δ2)	abct-F1		CAGAATAGGGGCGTTTTTCA	2338
		CR1-R		GCTTTGAAGGCTGGGTAGTG	
	PCR43 (Tn*1548*-like-2, -Δ1, and -Δ2)	abct-F2		CTGACCTTGTGGCACTTCAA	3774
		tnpU-R		GTGGCATCAATGACCAAGTG	
	PCR44 (Tn*1548*-like-1, ΔTn*6279*, and Tn*1548*-like-2, -Δ1, and -Δ2)	tnpU-F		TGGAAGAAAGACTCCGGCTA	790 / 2127
		armA-R		TGTTGCGACTCTTTCATTCG	
	PCR45 (Tn*1548*-like-1, ΔTn*6279*, and Tn*1548*-like-2, -Δ1, and -Δ2)	tnpD-F		GTCCTGGTTGGCAAAGGTTA	1015 / 1843
		msrE-R		TTTCCTGCTCCGTTATCACC	
	PCR46 (Tn*1548*-like-1, ΔTn*6279*, and Tn*1548*-like-2, -Δ1, and -Δ2)	pre-orfTn6257-F		GCGATGTTCCACGGTTTTAT	1929 / 4358
		IS26-R1		TCTGCCACTTCTTCACGTTG	
	PCR47 (Tn*6279* and Δ*Tn6279* downstream, linkage between Tn*1548*-like-1 and ΔTn*6279*, respectively, with the host chromosome)	repA-F	Internal, outwards	TCCAGTACTACGCCATGCAA	1426
		permease-R	External, inwards	AAGAACGTGCGGGTCTTATG	
	PCR48 (linkage between Tn*1548*-like-2, -Δ1 and -Δ2 and the host plasmid)	repA-F		TCCAGTACTACGCCATGCAA	2285
		res-R		GCGGTCTTGTTGATGCTGTA	
	PCR49 (host plasmid, adjacent to Tn*1548*-like-2, -Δ1 and -Δ2)	res-F		GTGCAGATCATGCTTTTTCG	2464
		CR2-R		GGACCGCAGTTGACTCTTTC	
	PCR50 (Tn*6279*, circular form)	repA-F	Internal, outwards	TCCAGTACTACGCCATGCAA	1909
		aphA1b-F (R)	Internal, outwards	CGACTGAATCCGGTGAGAAT	

TABLE S1. CONT. Primers and PCR assays.

PCR assay title	PCR assay code (genetic target)	Oligo Name	Oligo features	5'-3' sequence	Expected amplicon size(bp)
Genetic context of *armA*	PCR51 (Tn*1548*-like-1, circular form)	repA-F	Internal, outwards	TCCAGTACTACGCCATGCAA	578 / 1398
		intI1-R	Internal, outwards	GCCTTGATGTTACCCGAGAG	
	PCR52 (Tn*1548*-like-2, circular form)	repA-F	Internal, outwards	TCCAGTACTACGCCATGCAA	2273
		arr2-R	Internal, outwards	GCTCCATCAAGGCTGAAAAG	
	PCR53 (Tn*1548*-like-2-Δ1 and -Δ2, circular forms)	repA-F	Internal, outwards	TCCAGTACTACGCCATGCAA	1270 / 1347
		gst-R	Internal, outwards	AATCGCGGAACAATATCCAG	
	PCR54 (Tn*6279*, insretion site)	HPA2-F	External, inwards	GCTGCACAGTGGTTTTCTGA	655 / 1484
		permease-R	External, inwards	AAGAACGTGCGGGTCTTATG	
	PCR55 (Tn*6279*, insretion site)	araC-F	External, inwards	AATGCCAATGGGGATCATAA	Negative
		permease-R	External, inwards	AAGAACGTGCGGGTCTTATG	
Genetic context of *aphA1*	PCR58 (AbGR-12-like upstream)	AB57_1175-F	External, inwards	AGGAGATCTTCTTGGCAGTCA	2254
		TEM-D1-R	Internal, outwards	TTGCCGGGAAGCTAGAGTAA	
	PCR59 (linkage between TEM-D1 and Tn*1000*)	TEM-D1-F		GCGGCCAACTTACTTCTGAC	3238
		Tn1000-R		CGGTGTGACAAAACGCATAC	
	PCR60 (linkage between Tn*1000* and *aphA1*)	Tn1000-F		GCATAATATTCCCGCGCTTA	3416 / 3437 / 3612
		aphA1b-R		GGTTGCATTCGATTCCTGTT	
	PCR61 (*aphA1* downstream)	IS26-F		TGGTACTGGCGTAACCCTTC	1368 / 1543
		aphA1b-R		GGTTGCATTCGATTCCTGTT	
	PCR62 (*aphA1* upstream)	aphA1b-F		CGACTGAATCCGGTGAGAAT	1100
		IS26-R1		TCTGCCACTTCTTCACGTTG	
	PCR63 (linkage between *aphA1* and class 1 integron)	aphA1b-F		CGACTGAATCCGGTGAGAAT	5134 / 5323 / 5781
		aadA1-R		ACCAAGGCAACGCTATGTTC	
	PCR64 (linkage between IS*26* and class 1 integron)	IS26-F		TGGTACTGGCGTAACCCTTC	3319 / 4236 / 4425 / 4883
		aadA1-R		ACCAAGGCAACGCTATGTTC	
	PCR65 (class 1 integron)	intI1-F		TGCGTGTAAATCATCGTCGT	3056 / 3594 / 3695
		aadA1-R		ACCAAGGCAACGCTATGTTC	
	PCR66 (class 1 integron)	aadA1-F		TGATTTGCTGGTTACGGTGA	2297
		orf5-F (R)		TAGGAGCTCGGATCTCAGGA	
	PCR67 (linkage between IS*26* and class 1 integron)	IS26-F		TGGTACTGGCGTAACCCTTC	3776
		aadA1-F (R)		TGATTTGCTGGTTACGGTGA	
	PCR68 (linkage between class 1 integron and IS*26*)	aadA1-R (F)		ACCAAGGCAACGCTATGTTC	3725 / 4371
		Tn21-R		ATATACCCAATGCGCTGTCC	
	PCR69 (AbGR-12-like upstream)	AB57_1175-F	External, inwards	AGGAGATCTTCTTGGCAGTCA	4921
		Tn21-R	Internal, outwards	ATATACCCAATGCGCTGTCC	
	PCR70 (AbGR-12-like downstream)	Tn21-F	Internal, outwards	TACCGTGGCAGGAAGAAATC	2108
		AB57_1209-R	External, inwards	CATCTGCCATCCAGTTTGTG	

TABLE S1. CONT. Primers and PCR assays.

PCR assay title	PCR assay code (genetic target)	Oligo Name	Oligo features	5'-3' sequence	Expected amplicon size(bp)
Genetic context of *aphA1*	PCR71 (Tn*6020*, circular form)	aphA1b-circular-F	Internal, outwards	TGCATCATCAGGAGTACGGA	1620 / 1780 / 1947 / 1968 / 2143
		aphA1b-R	Internal, outwards	GGTTGCATTCGATTCCTGTT	
Genetic context of *aphA6*	PCR72 (Tn*aphA6* downstream)	aci6-plasmid-ST636-F	External, inwards	ACGGTTCACGAACTTTGACA	1471
		aphA6-R	Internal, outwards	TCGTGCTGGATTAGCAGATG	
	PCR73 (Tn*aphA6* upstream)	aphA6-F	Internal, outwards	GATGGCGACTGACCAATTTT	1484
		aci6-plasmid-ST636-R	External, inwards	CCTTAAGCTTTTCAGCTCATCAA	
	PCR74 (Tn*aphA6* downstream)	aci6-plasmid-ST25-F	External, inwards	TGAACAATGATACCTCGAAAGTT	1537 / 1792
		aphA6-R	Internal, outwards	TCGTGCTGGATTAGCAGATG	
	PCR75 (Tn*aphA6* upstream)	aphA6-F	Internal, outwards	GATGGCGACTGACCAATTTT	1633
		aci6-plasmid-ST25-R1	External, inwards	CTTGAGGAAGGGATGGTTGA	
	PCR76 (Tn*aphA6* upstream)	aphA6-F	Internal, outwards	GATGGCGACTGACCAATTTT	1490 / 2655 / 3745
		aci6-plasmid-ST25-R2	External, inwards	GCCTTTAAAGCAAAAACAAAGC	
	PCR77 (Aci6 plasmid)	aci6-plasmid-ST636-int-F1		TTTGCAATTATGGGCTCCAC	1044
		aci6-plasmid-ST636-int-R1		TACCCGACTCTTCAGGTGCT	
	PCR78 (Aci6 plasmid)	aci6-plasmid-ST636-int-F2		TTGCTTTAATCGGTGGTTCC	493
		aci6-plasmid-ST636-int-R2		TTATAGGCTGCACCGACACA	
	PCR79 (Tn*aphA6* circular form)	aphA6-F	Internal, outwards	GATGGCGACTGACCAATTTT	1442 / 1697
		aphA6-R	Internal, outwards	TCGTGCTGGATTAGCAGATG	
	PCR80 (Tn*aphA6* insretion site)	aci6-plasmid-ST636-F	External, inwards	ACGGTTCACGAACTTTGACA	423 / 1513 / 3498
		aci6-plasmid-ST636-R	External, inwards	CCTTAAGCTTTTCAGCTCATCAA	
	PCR81 (Tn*aphA6* insertion site)	aci6-plasmid-ST25-F	External, inwards	TGAACAATGATACCTCGAAAGTT	1728 / 3713
		aci6-plasmid-ST25-R1	External, inwards	CTTGAGGAAGGGATGGTTGA	
	PCR82 (Tn*aphA6* insertion site)	aci6-plasmid-ST25-F	External, inwards	TGAACAATGATACCTCGAAAGTT	1840 / 3825
		aci6-plasmid-ST25-R2	External, inwards	GCCTTTAAAGCAAAAACAAAGC	

TABLE S2. Quinolone, streptomycin, macrolide, and phenicol resistance features.

Isolate	Quinolone resistance chromosomal mutations		Streptomycin resistance genes	Macrolide resistance genes	Phenicol resistance genes			
	GyrA	ParC	*strA, strB*	*msr(E), mph(E)*	*cmlA7*	*catA1*	*catB8*	*floR*
A068	S83L	S80L	*strA*, *strB*	*msr(E)*, *mph(E)*	*cmlA7*	−	−	−
A069	S83L	S80L	*strA*, *strB*	*msr(E)*, *mph(E)*	−	−	−	−
A070	S83L	S80L	*strA*, *strB*	*msr(E)*, *mph(E)*	−	−	*catB8*	−
A071	S83L	S80L	*strA*, *strB*	*msr(E)*, *mph(E)*	−	−	*catB8*	−
A072	S83L	S80L	*strA*, *strB*	*msr(E)*, *mph(E)*	−	−	−	−
A074	S83L	S80L	−	−	−	*catA1*	−	−
A076	S83L	S80L	−	−	−	*catA1*	−	−
A077	S83L	S80L	*strA*, *strB*	−	−	−	−	−
A078	S83L	S80L	*strA*, *strB*	−	−	−	−	−
A079	S83L	S80L	*strA*, *strB*	*msr(E)*, *mph(E)*	−	−	−	−
A080	S83L	S80L	*strA*, *strB*	*msr(E)*, *mph(E)*	−	−	−	−
A082	S83L	S80L	−	−	−	*catA1*	−	*floR*
A084	S83L	S80L	*strA*, *strB*	−	−	−	−	−
A085	S83L	S80L	−	−	−	−	−	−
A086	S83L	S80L	*strA*, *strB*	*msr(E)*, *mph(E)*	−	−	−	−
A087	S83L	S80L	*strA*, *strB*	−	−	−	−	−
A089	S83L	S80L	−	*msr(E)*, *mph(E)*	−	−	−	−
A091	S83L	S80L	*strA*, *strB*	*msr(E)*, *mph(E)*	−	−	*catB8*	−
A092	S83L	S80L	*strA*, *strB*	*msr(E)*, *mph(E)*	*cmlA7*	−	−	−
A093	S83L	S80L	*strA*, *strB*	*msr(E)*, *mph(E)*	*cmlA7*	−	−	−
A094	S83L	S80L	*strA*, *strB*	*msr(E)*, *mph(E)*	−	−	−	−
A095	S83L	S80L	*strA*, *strB*	*msr(E)*, *mph(E)*	−	−	−	−
A096	S83L	S80L	*strA*, *strB*	*msr(E)*, *mph(E)*	*cmlA7*	−	−	−
A097	S83L	S80L	*strA*, *strB*	*msr(E)*, *mph(E)*	*cmlA7*	−	−	−
A099	S83L	S80L	−	*msr(E)*, *mph(E)*	−	*catA1*	−	−
A100	S83L	S80L	−	−	−	*catA1*	−	−
A101	S83L	S80L	*strA*, *strB*	−	−	−	−	−
A105	S83L	S80L	−	−	−	*catA1*	−	−

TABLE S3. Rifampicin, sulphonamide, and tetracycline resistance features.

Isolate	Rifampicin resistance genes	Sulphonamide resistance genes		Tetracycline resistance genes		
	arr-2	***sul1***	***sul2***	***tet(A)***	***tet(B)***	***tet(39)***
A068	*arr-2*	*sul1* (2 copies)	*sul2* (2 copies)	−	*tet(B)*	−
A069	−	*sul1*	*sul2* (2 copies)	−	*tet(B)*	−
A070	−	*sul1* (2 copies)	*sul2*	−	Δ*tet(B)*	−
A071	−	*sul1*	*sul2*	−	Δ*tet(B)*	−
A072	−	−	*sul2*	−	Δ*tet(B)*	−
A074	−	*sul1* (2 copies)	−	−	−	−
A076	−	*sul1* (2 copies)	−	*tet(A)*	−	−
A077	−	−	−	−	Δ*tet(B)*	−
A078	−	−	−	−	−	*tet(39)*
A079	−	−	−	−	Δ*tet(B)*	−
A080	−	−	−	−	Δ*tet(B)*	−
A082	−	*sul1* (2 copies)	−	*tet(A)*	−	−
A084	−	*sul1*	*sul2*	−	Δ*tet(B)*	−
A085	−	*sul1*	−	$tet(A)_{\mathrm{OIFC137}}$	−	−
A086	−	−	*sul2*	−	Δ*tet(B)*	−
A087	−	−	−	−	Δ*tet(B)*	−
A089	−	−	*sul2*	−	−	−
A091	−	*sul1*	−	−	Δ*tet(B)*	−
A092	*arr-2*	*sul1* (2 copies)	*sul2* (2 copies)	−	*tet(B)*	−
A093	*arr-2*	*sul1* (2 copies)	*sul2* (2 copies)	−	*tet(B)*	−
A094	−	*sul1*	*sul2* (2 copies)	−	*tet(B)*	−
A095	−	−	*sul2*	−	Δ*tet(B)*	−
A096	*arr-2*	*sul1* (2 copies)	*sul2* (2 copies)	−	*tet(B)*	−
A097	*arr-2*	*sul1* (2 copies)	*sul2* (2 copies)	−	*tet(B)*	−
A099	−	*sul1* (2 copies)	−	*tet(A)*	−	−
A100	−	*sul1* (2 copies)	−	*tet(A)*	−	−
A101	−	−	−	−	Δ*tet(B)*	−
A105	−	*sul1* (2 copies)	−	−	−	−

TABLE S4. Plasmid replicon types carried by the isolates, and the BioProject and BioSample accession numbers.

Isolate	Plasmid replicon types[a]							BioProject accession number	BioSample accession number
	GR1	GR2	GR6	GR7	GR8	GR10	GR14		
A068	−	Aci1	−	−	−	−	−	PRJNA282589	SAMN03571960
A069	−	−	−	−	−	−	−	PRJNA282600	SAMN03575971
A070	−	−	Aci6	−	Aci9	−	−	PRJNA282601	SAMN03575972
A071	−	−	−	−	−	−	−	PRJNA282602	SAMN03575974
A072	−	Aci1	Aci6	−	−	−	−	PRJNA282603	SAMN03576356
A074	−	Aci1	Aci6	−	−	−	−	PRJNA282604	SAMN03576358
A076	−	Aci1	−	−	−	−	p4AYE	PRJNA282605	SAMN03576359
A077	−	Aci1	−	−	−	−	−	PRJNA282606	SAMN03576361
A078	−	Aci1	−	−	−	−	−	PRJNA282607	SAMN03576362
A079	−	Aci1	−	−	−	−	−	PRJNA282608	SAMN03576363
A080	−	Aci1	−	−	−	−	−	PRJNA282609	SAMN03576364
A082	−	Aci1	Aci6	−	−	−	−	PRJNA282610	SAMN03576365
A084	−	Aci1	Aci6	−	−	−	−	PRJNA282611	SAMN03576366
A085	−	−	−	−	−	−	−	PRJNA282613	SAMN03576441
A086	p1S1	Aci1	−	−	−	−	−	PRJNA282614	SAMN03576442
A087	−	Aci1	−	−	−	−	−	PRJNA282615	SAMN03576445
A089	−	Aci1	−	−	−	−	−	PRJNA282616	SAMN03576446
A091	−	Aci1	Aci6	−	−	−	−	PRJNA282617	SAMN03576448
A092	−	Aci1	Aci6	−	−	−	−	PRJNA282618	SAMN03576449
A093	−	Aci1	−	−	−	−	−	PRJNA282619	SAMN03576505
A094	−	Aci1	Aci6	−	−	−	−	PRJNA282620	SAMN03576511
A095	−	Aci1	Aci6	−	−	−	−	PRJNA282622	SAMN03576512
A096	−	Aci1	Aci6	−	−	−	−	PRJNA282623	SAMN03577725
A097	−	Aci1	Aci6	−	−	−	−	PRJNA282626	SAMN03577726
A099	−	−	Aci6	−	−	AciX	−	PRJNA282627	SAMN03577727
A100	−	Aci1	−	p3S2	−	−	p4AYE	PRJNA282628	SAMN03577728
A101	−	Aci1	Aci6	−	−	−	−	PRJNA282640	SAMN03577729
A105	−	Aci1	Aci6	−	−	−	−	PRJNA282641	SAMN03577730

[a] According to the method described by **Bertini A, Poirel L, Mugnier PD, Villa L, Nordmann P, Carattoli A.** 2010. Characterization and PCR-based replicon typing of resistance plasmids in *Acinetobacter baumannii*. Antimicrob Agents Chemother **54:**4168–4177.

TABLE S5. Genetic structure and epidemiological data of plasmids carrying the *bla*$_{OXA-24}$ or *bla*$_{OXA-72}$ genes.

Plasmid	Comments	Size (bp)	Genetic structure	GenBank accession number	Referrence[a]
pABVA01	2000 / Varese, Italy / Int Clone II	8963	4 iterons, *repB*$_{pABVA01}$, *repA*$_{pABVA01}$, XerC/XerD-like sequence 1, *bla*$_{OXA-24}$, XerC/XerD-like sequence 2, *abkA*, *abkB*, *tonB*, *orf*$_{Septicolysin}$, XerC/XerD-like sequence 3, *fliD*, *orf1*, *orf2* (189 bp)	FM210331	DAndrea *et al*., 2009
pMMCU3	Spain	8964	4 iterons, *repB*$_{pABVA01}$, *repA*$_{pABVA01}$, XerC/XerD-like sequence 1, *bla*$_{OXA-24}$, XerC/XerD-like sequence 2, *abkA*, *abkB*, *tonB*, *orf*$_{Septicolysin}$, XerC/XerD-like sequence 3, *fliD*, *orf1*, *orf2* (189 bp)	GQ904227	Merino *et al*., 2010
pA077	2013 / Skåne, Sweden / ST2	8963	4 iterons, *repB*$_{pABVA01}$, *repA*$_{pABVA01}$, XerC/XerD-like sequence 1, *bla*$_{OXA-24}$, XerC/XerD-like sequence 2, *abkA*, *abkB*, *tonB*, *orf*$_{Septicolysin}$, XerC/XerD-like sequence 3, *fliD*, *orf1*, *orf2* (189 bp)	This study	This study
pA087	2013 / Västra Götaland, Sweden / ST2	8963	4 iterons, *repB*$_{pABVA01}$, *repA*$_{pABVA01}$, XerC/XerD-like sequence 1, *bla*$_{OXA-24}$, XerC/XerD-like sequence 2, *abkA*, *abkB*, *tonB*, *orf*$_{Septicolysin}$, XerC/XerD-like sequence 3, *fliD*, *orf1*, *orf2* (189 bp)	This study	This study
pAB-NCGM253	2012 / Japan	8970	4 iterons, *repB*$_{pAB0057}$, *repA*$_{pAB0057}$, XerC/XerD-like sequence 1, *bla*$_{OXA-72}$, XerC/XerD-like sequence 2, *abkA*, *abkB*, *tonB*, *orf*$_{Septicolysin}$, XerC/XerD-like sequence 3, *fliD*, *orf1*, *orf2* (339 bp)	AB823544	Tada *et al.*, 2014
pA074	2012 / Skåne, Sweden / ST636	9831	4 iterons, *repB*$_{pAB0057}$, *repA*$_{pAB0057}$, XerC/XerD-like sequence 1, *bla*$_{OXA-72}$, IS*Aba31*, XerC/XerD-like sequence 2, *abkA*, *abkB*, *tonB*, *orf*$_{Septicolysin}$, XerC/XerD-like sequence 3, *fliD*, *orf1*, *orf2* (339 bp)	This study	This study
pA078	2013 / Västra Götaland, Sweden / ST23	9831	4 iterons, *repB*$_{pAB0057}$, *repA*$_{pAB0057}$, XerC/XerD-like sequence 1, *bla*$_{OXA-72}$, IS*Aba31*, XerC/XerD-like sequence 2, *abkA*, *abkB*, *tonB*, *orf*$_{Septicolysin}$, XerC/XerD-like sequence 3, *fliD*, *orf1*, *orf2* (339 bp)	This study	This study
pA100	2013 / Skåne, Sweden / ST1	15166	4 iterons, *repB*$_{pAB0057}$, *repA*$_{pAB0057}$, XerC/XerD-like sequence 1, *bla*$_{OXA-72}$, IS*Aba31*, XerC/XerD-like sequence 2, *abkA*, *abkB*, *tonB*, *orf*$_{Septicolysin}$, XerC/XerD-like sequence 3, *orf*, *mobA*, *rep3*, XerC/XerD-like sequence 4, *emrE*, *orf*$_{Plasmid\ mobilization}$, XerC/XerD-like sequence 5, *fliD*, *orf1*, *orf2* (339 bp)	This study	This study
pA105	2013 / Stockholm, Sweden / ST636	9830	4 iterons, *repB*$_{pAB0057}$, *repA*$_{pAB0057}$, XerC/XerD-like sequence 1, *bla*$_{OXA-72}$, IS*Aba31*, XerC/XerD-like sequence 2, *abkA*, *abkB*, *tonB*, *orf*$_{Septicolysin}$, XerC/XerD-like sequence 3, *fliD*, *orf1*, *orf2* (339 bp)	This study	This study

[a] **D'Andrea MM, Giani T, D'Arezzo S, Capone A, Petrosillo N, Visca P, Luzzaro F, Rossolini GM.** 2009. Characterization of pABVA01, a plasmid encoding the OXA-24 carbapenemase from Italian isolates of *Acinetobacter baumannii*. Antimicrob Agents Chemother **53:**3528–3533.

Merino M, Acosta J, Poza M, Sanz F, Beceiro A, Chaves F, Bou G. 2010. OXA-24 carbapenemase gene flanked by XerC/XerD-like recombination sites in different plasmids from different *Acinetobacter* species isolated during a nosocomial outbreak. Antimicrob Agents Chemother **54:**2724–2727.

Tada T, Miyoshi-Akiyama T, Shimada K, Shimojima M, Kirikae T. 2014. Dissemination of 16S rRNA methylase ArmA-producing *Acinetobacter baumannii* and emergence of OXA-72 carbapenemase coproducers in Japan. Antimicrob Agents Chemother **58:**2916–2920.

TABLE S6. Numeration of the GenBank variants of *bla*$_{ADC}$.

Variant[a]	GenBank accession number		Species of first identification	NCBI reference sequence, length[b]	Reference[c]	Previous designations
	Protein	Nucleotide				
ADC-1	CAB77444	AJ009979	*Acinetobacter baumannii*	WP_004714775	Bou & Martinez-Beltran, 2000	ADC-NIPH 1362
ADC-2	AAO43172	AY177427	*Oligella urethralis*	WP_004746565	Mammeri *et al*., 2003	ADC-NIPH 1734
ADC-3	AAO59456	AY178995	*A. baumannii*	−	Mammeri *et al*., 2003	−
ADC-4	AAO59457	AY178996	*A. baumannii*	−	Mammeri *et al*., 2003	−
ADC-5	CAE00827	AJ575184	*Acinetobacter pittii*	WP_038405930	Beceiro *et al*., 2004	−
ADC-6	AAR13676	AY325306	*A. baumannii*	WP_017725267	Segal *et al*., 2004	−
ADC-7	AAT70411	AY648950	*A. baumannii*	−	Hujer *et al*., 2005	−
ADC-10	ABI18382	DQ883640	*A. baumannii*	−	Hujer *et al*., not published	−
ADC-11	ADG46039	GU591983; CU459141	*A. baumannii*	WP_001211205	Rodriguez-Martinez *et al*., 2010; Fournier *et al*., 2006	−
ADC-12	CAK95249	AM283529	*A. pittii*	−	Beceiro *et al*., 2009	−
ADC-13	CAK95248	AM283528	*A. pittii*	−	Beceiro *et al*., 2009	−
ADC-14	CAK95247	AM283527	*A. pittii*	−	Beceiro *et al*., 2009	−
ADC-15	CAK95246	AM283526	*A. pittii*	−	Beceiro *et al*., 2009	−
ADC-16	CAK95245	AM283525	*A. pittii*	−	Beceiro *et al*., 2009	−
ADC-17	CAK95244	AM283524	*A. pittii*	−	Beceiro *et al*., 2009	−
ADC-18	CAK95243	AM283523	*A. pittii*	WP_002118772	Beceiro *et al*., 2009	−
ADC-19	CAK95242	AM283522	*A. pittii*	−	Beceiro *et al*., 2009	−
ADC-20	CAK95241	AM283521	*A. pittii*	−	Beceiro *et al*., 2009	−
ADC-21	CAK95240	AM283520	*A. pittii*	−	Beceiro *et al*., 2009	−
ADC-22	CAK95239	AM283519	*A. pittii*	−	Beceiro *et al*., 2009	−
ADC-23	CAK95238	AM283518	*A. pittii*	−	Beceiro *et al*., 2009	−
ADC-24	CAK95237	AM283517	*A. pittii*	−	Beceiro & Bou., not published	ADC-19
ADC-25	ABK34773	EF016355	*A. baumannii*	WP_001211217	Zong *et al*., 2008	ADC-NIPH 528
ADC-26	ADG46043	GU591987	*A. baumannii*	WP_001211238	Rodriguez-Martinez *et al*., 2010	ADC-NIPH 146
ADC-29	ACC66195	EU604835	*A. baumannii*	−	Chiu *et al*., not published	−
ADC-30	ADG46041	GU591985	*A. baumannii*	WP_001211218	Rodriguez-Martinez *et al*., 2010	ADC-NIPH 2061
ADC-38	ACC95873	EU652243	*A. baumannii*	−	Bogaerts *et al*. 2008	−
ADC-39	ACC95874	EU652244	*A. baumannii*	−	Bogaerts *et al*. 2008	−
ADC-41	ACN62070	FJ744160	*A. pittii*	−	Huang *et al*., 2010	−
ADC-42	ACN62071	FJ744161	*A. pittii*	−	Huang *et al*., 2010	−
ADC-43	ACN62072	FJ744162	*A. pittii*	WP_032055358	Huang *et al*., 2010	−
ADC-44	ACN62073	FJ744163	*A. pittii*	−	Huang *et al*., 2010	−
ADC-50	ADG46038	GU591982	*A. baumannii*	WP_031965243	Rodriguez-Martinez *et al*., not published	−
ADC-51	ADG46040	GU591984	*A. baumannii*	−	Rodriguez-Martinez *et al*., 2010	−
ADC-52	ADG46042	GU591986	*A. baumannii*	WP_001211232	Rodriguez-Martinez *et al*., 2010	−
ADC-53	ADG46044	GU591988	*A. baumannii*	−	Rodriguez-Martinez *et al*., 2010	−
ADC-54	ADK35761	HM437231	*A. baumannii*	−	Bogaerts *et al*., 2010	−

TABLE S6. CONT. Numeration of the GenBank variants of *bla*$_{ADC}$.

Variant[a]	GenBank accession number		Species of first identification	NCBI reference sequence, length[b]	Reference[c]	Previous designations
	Protein	Nucleotide				
ADC-56	AEL30570	JF265067	*A. baumannii*	WP_031973850	Tian *et al*., 2011	–
ADC-57	ADO51072	HQ258925	*A. baumannii*	WP_001211226	Revathi *et al*., 2013	–
ADC-58	AFG25594	JQ319653	*A. baumannii*	–	Zhang, not published	–
ADC-59	AFG25595	JQ319654	*A. baumannii*	–	Zhang, not published	–
ADC-60	AFH53180	JQ692087	*A. baumannii*	–	Huang, not published	–
ADC-61	AFI56570	JQ753702	*A. baumannii*	WP_033503051	Zhou, not published	–
ADC-62	AFK24475	JQ867374	*A. baumannii*	–	Wang, not published	–
ADC-63	AFM80040	JQ911781	*A. baumannii*	–	Zhang, not published	–
ADC-64	AFM80041	JQ911782	*A. baumannii*	–	Zhang, not published	–
ADC-65	AFP73417	JX109941	*A. baumannii*	385 amino acid	Ling *et al*., 2013	–
ADC-66	AFP73418	JX109942	*A. baumannii*	–	Ling *et al*., 2013	–
ADC-67	AFP89364	JX169789	*A. baumannii*	–	Xiao-min *et al*., 2014	ADC-57 (AEZ36052)
ADC-68	AGL39360	KC866352	*A. baumannii*	–	Lee *et al*., 2014 (as a poster); Jeon *et al*., 2014	–
ADC-72	AIL90389	KJ885607	*Acinetobacter calcoaceticus*	WP_017480710	Sun *et al*., 2014	–
ADC-73	ALA14808	KP881233	*A. baumannii*	WP_001211219	This study (isolate A072)	–
ADC-74	ALA14809	KP881234	*A. baumannii*	WP_001211203	This study (isolate A074)	–
ADC-75	ALA14810	KP881235	*A. baumannii*	–	This study (isolate A076)	–
ADC-76	ALA14811	KP881236	*A. baumannii*	WP_001211237	This study (isolate A078, intrinsic)	ADC-NIPH 335
ADC-77	ALA14812	KP881237	*A. baumannii*	–	This study (isolate A078, acquired)	–
ADC-78	ALA14813	KP881238	*A. baumannii*	WP_057691006	This study (isolate A082)	–
ADC-79	ALA14814	KP881239	*A. baumannii*	WP_001159760	This study (isolate A085)	–
ADC-80	ALA14815	KP881240	*A. baumannii*	WP_029424536	This study (isolate A099)	–
ADC-81	ALA14816	KP881241	*A. baumannii*	388 amino acid	This study (isolate A100)	–
ADC-82	AAV32519	AY758396	*A. baumannii*	–	Heritier *et al*., 2006	–
ADC-83	ABO38124	EF433777	*A. baumannii*	–	Huang *et al*., 2007 unpublished	–
ADC-84	ABV21800	EU118261	*A. baumannii*	WP_001211220, 384 amino acid	Bratu *et al*., 2008	–
ADC-85	ABV21801	EU118262	*A. baumannii*	–	Bratu *et al*., 2008	–
ADC-86	ABV21802	EU118263	*A. baumannii*	–	Bratu *et al*., 2008	–
ADC-87	ETY67158	AZNQ01000099	*A. baumannii*	384 amino acid	Dent *et al*., 2010	–
ADC-88	ADX04315	CP001921	*A. baumannii*	WP_001211223	Park *et al*., 2011	–
ADC-89	AFU38919	CP003856	*A. baumannii*	–	Liou *et al*., 2012	–
ADC-90	WP_017816757	NZ_AOLU01000048	*A. baumannii*	WP_017816757	Ho *et al*., 2013	–
ADC-91	ELW88222	AMGA01000054	*A. baumannii*	WP_002157727	Harkins *et al*., 2013 unpublished	–
ADC-92	ENW75976	APRG01000011	*A. baumannii*	WP_001211227	Perichon *et al*., 2014	ADC-CIP 70–34[T]
ADC-93	ENU51112	APOQ01000006	*A. baumannii*	WP_004712857	Perichon *et al*., 2014	ADC-NIPH 1669

TABLE S6. CONT. Numeration of the GenBank variants of bla_{ADC}.

Variant[a]	GenBank accession number		Species of first identification	NCBI reference sequence, length[b]	Reference[c]	Previous designations
	Protein	Nucleotide				
ADC-94	ENV26641	APPL01000015	*A. baumannii*	WP_002126587	Perichon *et al.*, 2014	ADC-NIPH 190
ADC-95	ENW36647	APQV01000007	*A. baumannii*	WP_005109685	Perichon *et al.*, 2014	ADC-NIPH 201
ADC-96	ENW46489	APQY01000003	*A. baumannii*	WP_005123276	Perichon *et al.*, 2014	ADC-NIPH 329
ADC-97	ENV30802	APPM01000011	*A. baumannii*	WP_004840559	Perichon *et al.*, 2014	ADC-NIPH 60
ADC-98	ENW51893	APQZ01000007	*A. baumannii*	WP_005128228	Perichon *et al.*, 2014	ADC-NIPH 601
ADC-99	ENU68675	APOV01000029	*A. baumannii*	WP_004739487	Perichon *et al.*, 2014	ADC-NIPH 615
ADC-100	ENW51227	APRA01000004	*A. baumannii*	WP_005131186	Perichon *et al.*, 2014	ADC-NIPH 67
ADC-101	ENW72863	APRE01000031	*A. baumannii*	WP_005138362	Perichon *et al.*, 2014	ADC-NIPH 80
ADC-102	ENW00696	APQI01000003	*A. calcoaceticus*	WP_005046018	Perichon *et al.*, 2014	ADC-CIP 81–8[T]
ADC-103	ENU07956	APOE01000009	*A. calcoaceticus*	WP_004643536	Perichon *et al.*, 2014	ADC-NIPH 13
ADC-104	ENV92309	APQH01000006	*A. calcoaceticus*	WP_005039111	Perichon *et al.*, 2014	ADC-ANC 3680
ADC-105	ENV41121	APPP01000013	*Acinetobacter nosocomialis*	WP_004886093	Perichon *et al.*, 2014	ADC-NIPH 386
ADC-106	ENU48760	APOP01000002	*A. nosocomialis*	WP_004707701	Perichon *et al.*, 2014	ADC-NIPH 2119[T]
ADC-107	ENW11417	APQN01000012	*A. pittii*	WP_005068074	Perichon *et al.*, 2014	ADC-ANC 3678
ADC-108	ENU43147	APOO01000021	*Acinetobacter* gen. sp. "Close to 13TU"	WP_004700205	Perichon *et al.*, 2014	ADC-NIPH 973
ADC-109	ENX43770	APSC01000009	*Acinetobacter* gen. sp. "Between 1 and 3"	WP_005307218	Perichon *et al.*, 2014	ADC-NIPH 542
ADC-110	ENV03983	APPF01000018	*Acinetobacter* gen. sp. "Between 1 and 3"	WP_004790939	Perichon *et al.*, 2014	ADC-NIPH 817
ADC-111	EOQ64883	APQJ01000005	*A. calcoaceticus*-like	WP_016137488	Perichon *et al.*, 2014	ADC-ANC 3811
ADC-112	EOQ71234	APQM01000001	*A. pittii*-like	WP_016140427	Perichon *et al.*, 2014	ADC-ANC 4050
ADC-113	EOQ73533	APQO01000006	*A. pittii*-like	WP_016146025	Perichon *et al.*, 2014	ADC-ANC 4052
ADC-114	EXS60093	JFYI01000005	*A. baumannii*	WP_032039838	Harris *et al.*, unpublished	–
ADC-115	EYS55294	JHPF01000005	*A. baumannii*	WP_001211209	Harris *et al.*, unpublished	–
ADC-116	EXD64655	JEZV01000049	*A. baumannii*	WP_032062810	Harris *et al.*, unpublished	–
ADC-117	ETP95102	AYGO01000141	*A. baumannii*	WP_031980335	Wright *et al.*,	–
ADC-118	WP_033502167	NZ_AGSU01000096	*A. baumannii*	WP_033502167	Liou *et al.*, unpublished	–
ADC-119	WP_001211214	NZ_AMIJ01000141	*A. baumannii*	WP_001211214	Sahl *et al.*, unpublished	–
ADC-120	KHY08585	JWWE03000102	*A. baumannii*	WP_039270258	Adams *et al.*, unpublished	–
ADC-121	KHV30477	JWSI03000106	*A. baumannii*	WP_039258389	Adams *et al.*, 2015 unpublished	–
ADC-122	KJC71195	JZCF01000044	*A. baumannii*	WP_044718369	Adams *et al.*, 2015 unpublished	–

TABLE S6. CONT. Numeration of the GenBank variants of *bla*$_{ADC}$.

Variant[a]	GenBank accession number: Protein	GenBank accession number: Nucleotide	Species of first identification	NCBI reference sequence, length[b]	Reference[c]	Previous designations
ADC-401	CAL25116	AM293332	*Acinetobacter baylyi*	WP_004923134, 397 amino acid	Beceiro *et al*., 2007	ADC-8, ADC-CIP 107474[T]
ADC-501	ENW07270	APQL01000005	*Acinetobacter beijerinckii*	WP_005060342, 395 amino acid	Perichon *et al*., 2014	ADC-CIP 110307[T]
ADC-502	ENW06097	APQK01000009	*A. beijerinckii*	WP_039909284, 395 amino acid	Perichon *et al*., 2014	ADC-ANC 3835
ADC-503	ENW20454	APQQ01000012	*Acinetobacter haemolyticus*	WP_005087351, 395 amino acid	Perichon *et al*., 2014	ADC-CIP 64–3[T]
ADC-504	ENW22241	APQR01000003	*A. haemolyticus*	WP_005088798, 395 amino acid	Perichon *et al*., 2014	ADC-NIPH 261
ADC-505	ENV65577	APPX01000035	*Acinetobacter junii*	WP_026057384, 397 amino acid	Perichon *et al*., 2014	ADC-CIP 64–5[T]
ADC-506	ENV51174	APPS01000043	*A. junii*	WP_042106493, 395 amino acid	Perichon *et al*., 2014	ADC-CIP 107470
ADC-507	ENV64403	APPW01000004	*A. junii*	WP_051067828, 395 amino acid	Perichon *et al*., 2014	ADC-NIPH 182
ADC-508	ENV74627	APQA01000028	*Acinetobacter ursingii*	WP_004986092, 401 amino acid	Perichon *et al*., 2014	ADC-CIP 107286[T]
ADC-509	ENV78143	APQC01000023	*A. ursingii*	WP_005005518, 401 amino acid	Perichon *et al*., 2014	ADC-ANC 3649
ADC-510	ENX45677	APQB01000030	*A. ursingii*	WP_004999532, 401 amino acid	Perichon *et al*., 2014	ADC-NIPH 706
ADC-511	ENV38300	APPO01000008	*Acinetobacter venetianus*	WP_004878176, 392 amino acid	Perichon *et al*., 2014	ADC-CIP 110063[T]
ADC-512	ENU28809	APOK01000047	*Acinetobacter* gen. sp. 6	WP_004673603, 395 amino acid	Perichon *et al*., 2014	ADC-CIP A165
ADC-513	ENW97918	APRM01000005	*Acinetobacter* gen. sp. 6	WP_005194146, 395 amino acid	Perichon *et al*., 2014	ADC-NIPH 298
ADC-514	ENX35907	APRZ01000007	*Acinetobacter* gen. sp. 13BJ/14TU	WP_032878960, 395 amino acid	Perichon *et al*., 2014	ADC-NIPH 1859
ADC-515	ENX18440	APRT01000003	*Acinetobacter* gen. sp. 13BJ/14TU	WP_026040157, 395 amino acid	Perichon *et al*., 2014	ADC-CIP 64–2
ADC-516	ENX09334	APRR01000006	*Acinetobacter* gen. sp. 14BJ	WP_005228083, 395 amino acid	Perichon *et al*., 2014	ADC-NIPH 1847
ADC-517	ENX39917	APSA01000003	*Acinetobacter* gen. sp. 14BJ	WP_005282242, 395 amino acid	Perichon *et al*., 2014	ADC-NIPH 3623 (corrected)
ADC-518	ENX56225	APSD01000035	*Acinetobacter* gen. sp. 16	WP_005317155, 395 amino acid	Perichon *et al*., 2014	ADC-ANC 3880
ADC-519	ENV10055	APPH01000006	*Acinetobacter* gen. sp. 16	WP_004803441, 395 amino acid	Perichon *et al*., 2014	ADC-CIP 56–2
ADC-520	ENX60630	APRN01000033	*Acinetobacter* gen. sp. 16	WP_005201690, 395 amino acid	Perichon *et al*., 2014	ADC-CIP 70–18
ADC-521	ENW94247	APRL01000010	*Acinetobacter* gen. sp. 17	WP_005186110, 395 amino acid	Perichon *et al*., 2014	ADC-ANC 4105
ADC-522	ENX53738	APRO01000007	*Acinetobacter* gen. sp. 17	WP_005209612, 395 amino acid	Perichon *et al*., 2014	ADC-NIPH 1867
ADC-523	ENW98345	APRP01000033	*Acinetobacter* Taxon 18	WP_005219147, 388 amino acid	Perichon *et al*., 2014	ADC-ANC 3862
ADC-524	ENU27798	APOJ01000016	*Acinetobacter* Taxon 18	WP_004659736, 388 amino acid	Perichon *et al*., 2014	ADC-NIPH 236
ADC-525	WP_050041209	NZ_LACJ01000004	*Acinetobacter parvus*	WP_050041209, 388 amino acid	Pedron *et al*., 2015 unpublished	–

TABLE S6. CONT. Numeration of the GenBank variants of *bla*$_{ADC}$.

Variant[a]	GenBank accession number		Species of first identification	NCBI reference sequence, length[b]	Reference[c]	Previous designations
	Protein	Nucleotide				
ADC-526	ESK55746	AYEV01000014	*Acinetobacter tjernbergiae*	WP_023274629, 388 amino acid	Cerqueira *et al.*, 2013 unpublished	–
ADC-527	ENU21567	APOI01000030	*Acinetobacter* Taxon 19	WP_004656962, 395 amino acid	Perichon *et al.*, 2014	ADC-NIPH 809
ADC-528	ENU93346	APPC01000015	*Acinetobacter* Taxon 20	WP_004776385, 395 amino acid	Perichon *et al.*, 2014	ADC-NIPH 758
ADC-529	ENX19523	APRW01000016	*Acinetobacter* Taxon 20	WP_005260284, 395 amino acid	Perichon *et al.*, 2014	ADC-NIPH 2168
ADC-530	ENW83258	APRH01000012	*Acinetobacter* Taxon 21	WP_005147866, 395 amino acid	Perichon *et al.*, 2014	ADC-ANC 3929 (corrected)
ADC-531	ENX38460	APSB01000022	*Acinetobacter* Taxon 22	WP_005297698, 395 amino acid	Perichon *et al.*, 2014	ADC-NIPH 2100
ADC-601	ENV90446	APQG01000050	*Acinetobacter bereziniae*	WP_005034629, 390 amino acid	Perichon *et al.*, 2014	ADC-CIP 70–12[T]
ADC-602	ENV20683	APPK01000046	*A. bereziniae*	WP_004832033, 390 amino acid	Perichon *et al.*, 2014	ADC-NIPH 3
ADC-603	ENU57495	APOS01000032	*Acinetobacter guillouiae*	WP_004723463, 391 amino acid	Perichon *et al.*, 2014	ADC-CIP 63–46[T]
ADC-604	ENV14942	APPJ01000016	*A. guillouiae*	WP_004823669, 391 amino acid	Perichon *et al.*, 2014	ADC-NIPH 991
ADC-701	ENV73166	APPZ01000007	*Acinetobacter johnsonii*	WP_004983048, 388 amino acid	Perichon *et al.*, 2014	ADC-ANC 3681
ADC-702	ENU99039	APPE01000056	*Acinetobacter* gen. sp. 15TU	WP_004783170, 386 amino acid	Perichon *et al.*, 2014	ADC-NIPH 899
ADC-703	ENU21210	APOH01000008	*Acinetobacter* Taxon 26	WP_004650432, 388 amino acid	Perichon *et al.*, 2014	ADC-ANC 3994

[a] The variants were divided into 5 groups according to the similarities (>69%) among their amino acid sequences. Numeration started at 1 (first group), 401 (second group), 501 (third group), 601 (fourth group), and 701 (fifth group).

[b] If not stated, the length is 383 amino acid.

[c] The list of references for this table is presented below:

Bou, G., and J. Martinez-Beltran. 2000. Cloning, nucleotide sequencing, and analysis of the gene encoding an AmpC β-lactamase in *Acinetobacter baumannii*. Antimicrob. Agents Chemother. 44:428–32.

Mammeri, H., L. Poirel, N. Mangeney, and P. Nordmann. 2003. Chromosomal integration of a cephalosporinase gene from *Acinetobacter baumannii* into *Oligella urethralis* as a source of acquired resistance to β-lactams. Antimicrob. Agents Chemother. 47:1536–42.

Beceiro, A., L. Dominguez, A. Ribera, J. Vila, F. Molina, R. Villanueva, J. M. Eiros, and G. Bou. 2004. Molecular characterization of the gene encoding a new AmpC β-lactamase in a clinical strain of *Acinetobacter* genomic species 3. Antimicrob. Agents Chemother. 48:1374–8.

Segal, H., E. C. Nelson, and B. Gay Elisha. 2004. Genetic environment and transcription of *ampC* in an *Acinetobacter baumannii* clinical isolate. Antimicrob. Agents Chemother. 48:612–4.

Hujer, K. M., N. S. Hamza, A. M. Hujer, F. Perez, M. S. Helfand, C. R. Bethel, J. M. Thomson, V. E. Anderson, M. Barlow, L. B. Rice, F. C. Tenover, and R. A. Bonomo. 2005. Identification of a new allelic variant of the *Acinetobacter baumannii* cephalosporinase, ADC-7 β-lactamase: defining a unique family of class C enzymes. Antimicrob. Agents Chemother. 49:2941–8.

Beceiro A, Perez-Llarena FJ, Perez A, Tomas Mdel M, Fernandez A, et al. 2007. Molecular characterization of the gene encoding a new AmpC beta-lactamase in *Acinetobacter baylyi*. J Antimicrob Chemother 59: 996–1000.

Rodríguez-Martínez JM, Poirel L, Nordmann P. 2010. Genetic and functional variability of AmpC-type β-lactamases from *Acinetobacter baumannii*. Antimicrob. Agents Chemother. 54:4930–3.

Fournier PE, Vallenet D, Barbe V, Audic S, Ogata H, Poirel L, Richet H, Robert C, Mangenot S, Abergel C, Nordmann P, Weissenbach J, Raoult D, Claverie JM. 2006. Comparative genomics of multidrug resistance in *Acinetobacter baumannii*. PLoS Genet. 2:e7.

Beceiro A, Perez A, Fernandez-Cuenca F, Martinez-Martinez L, Pascual A, Vila J et al. 2009. Genetic variability among *ampC* genes from *Acinetobacter* genomic species 3. Antimicrob Agents Chemother. 53: 1177–84.

Zong Z, Lu X, Valenzuela JK, Partridge SR, and Iredell J. 2008. An outbreak of carbapenem-resistant *Acinetobacter baumannii* producing OXA-23 carbapenemase in western China. Int J Antimicrob Agents. 31:50–4.

Bogaerts, P., G. Cuzon, T. Naas, C. Bauraing, A. Deplano, B. Lissoir, P. Nordmann, and Y. Glupczynski. 2008. Carbapenem-resistant *Acinetobacter baumannii* isolates expressing the $bla_{\mathrm{OXA-23}}$ gene associated with IS*Aba4* in Belgium. Antimicrob. Agents Chemother. 52:4205–6.

Huang LY, Lu PL, Chen TL, Chang FY, Fung CP, Siu LK. 2010. Molecular characterization of ß-lactamase genes and their genetic structures in *Acinetobacter* genospecies 3 isolates in Taiwan. Antimicrob Agents Chemother 54:2699-703.

Bogaerts P, Naas T, El Garch F, Cuzon G, Deplano A, Delaire T, et al. 2010. GES extended-spectrum beta-lactamases in *Acinetobacter baumannii* isolates in Belgium. Antimicrob Agents Chemother. 54(11):4872–8.

Tian GB, Adams-Haduch JM, Taracila M, Bonomo RA, Wang HN, Doi Y. 2011. Extended-spectrum AmpC cephalosporinase in *Acinetobacter baumannii*: ADC-56 confers resistance to cefepime. Antimicrob. Agents Chemother. 55:4922–5.

Zhou Y, Teng SJ, Yang L, Li SB, Xu Y. 2015. A novel variant of the β-lactamase ADC-61 gene in multi-drug resistant *Acinetobacter baumannii*. Genet Mol Res 14:7092–100.

Ling Yue-Ming, Lan Xiao-peng, and Wang Jian-fu. 2013. Emergence of novel variants of β-lactamase gene $bla_{\mathrm{ADC-65}}$ and $bla_{\mathrm{ADC-66}}$ in pandrug-resistant *Acinetobacter baumannii*. Chinese Journal of Antibiotics 2:155-8 (article in Chinese).

Xiao-Min X, You-Fen F, Wei-Yun F, Zu-Huang M, Xing-Bei W. 2014. Antibiotic resistance determinants of a group of multidrug-resistant *Acinetobacter baumannii* in China. J Antibiot (Tokyo) 67(6):439-44.

J. H. Jeon, M.-K. Hong, J. H. Lee, J. J. Lee, K. S. Park, A. M. Karim, J. Y. Jo, J. H. Kim, K. S. Ko, L.-W. Kang and S. H. Lee. 2014. Structure of ADC-68, a novel carbapenem-hydrolyzing class C extended-spectrum β-lactamase isolated from *Acinetobacter baumannii*. Acta Cryst D70:2924–36.

Sun, Y., Liu, Q., Chen, S., Song, Y., Liu, J., Guo, X., Zhu, L., Ji, X., Xu, L., Zhou, W., Qian, J., Feng, S. 2014. Characterization and plasmid elimination of NDM-1-producing *Acinetobacter calcoaceticus* from China. PLoS ONE, 9:e106555.

Revathi G, Siu LK, Lu PL, Huang LY. 2013. First report of NDM-1-producing *Acinetobacter baumannii* in East Africa. Int J Infect Dis 17:e1255–e1258.

Heritier C., Poirel L., Nordmann P. 2006. Cephalosporinase over-expression resulting from insertion of IS*Aba1* in *Acinetobacter baumannii*. Clin. Microbiol. Infect. 12:123–130.

Bratu S, Landman D, Martin DA, Georgescu C, Quale J. 2008. Correlation of antimicrobial resistance with beta-lactamases, the OmpA-like porin, and efflux pumps in clinical isolates of *Acinetobacter baumannii* endemic to New York City. Antimicrob Agents Chemother 52:2999–3005.

Dent LL, Marshall DR, Pratap S, Hulette RB. 2010. Multidrug resistant *Acinetobacter baumannii*: a descriptive study in a city hospital. BMC Infect Dis. 10:196.

Park JY, Kim S, Kim SM, Cha SH, Lim SK, and Kim J. 2011. Complete genome sequence of multidrug-resistant *Acinetobacter baumannii* strain 1656-2, which forms sturdy biofilm. J Bacteriol. 193:6393–4.

Liou ML, Liu CC, Lu CW, Hsieh MF, Chang KC, Kuo HY, Lee CC, Chang CT, Yang CY, Tang CY. 2012. Genome sequence of *Acinetobacter baumannii* TYTH-1. J Bacteriol. 194:6974.

Ho AYM, Chow K-H, Law PYT, Tse H, Ho P-L. 2013. Draft genome sequences of two multidrug-resistant *Acinetobacter baumannii* strains of sequence type ST92 and ST96. Genome Announcements. 1(3):e00296-13.

Périchon B, Goussard S, Walewski V, Krizova L, Cerqueira G, Murphy C, et al. 2014. Identification of 50 class D β-lactamases and 65 *Acinetobacter*-derived cephalosporinases in *Acinetobacter* spp. Antimicrob Agents Chemother. 58(2):936–49.

Wright MS, Haft DH, Harkins DM, Perez F, Hujer KM, Bajaksouzian S, Benard MF, Jacobs MR, Bonomo RA, Adams MD. 2014. New insights into dissemination and variation of the health care-associated pathogen *Acinetobacter baumannii* from genomic analysis. mBio 5:e00963-13.

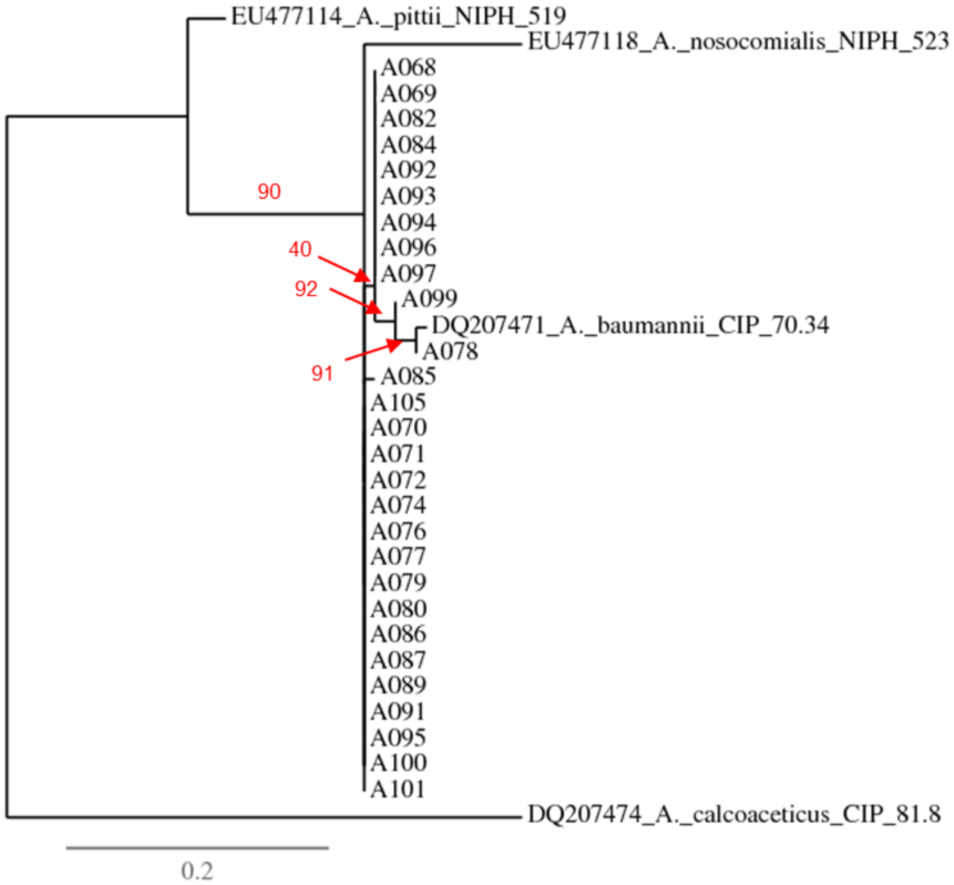

FIGURE S1. Species identification of the isolates using partial *rpoB* sequence analysis. The phylogenetic tree was constructed based on pairwise nucleotide sequence alignments of 352 bp of the *rpoB* gene of 28 clinical isolates of *Acinetobacter baumannii*, in comparison to the corresponding sequences of the *Acinetobacter calcoaceticus* CIP 81.8 (DQ207474), *Acinetobacter pittii* NIPH 519 (EU477114), *Acinetobacter nosocomialis* NIPH 523 (EU477118), and *A. baumannii* CIP 70.34 (DQ207471) reference strains. MUSCLE, Gblocks, PhyML, and TreeDyn were used for nucleotide alignment and tree construction. One hundred bootstraps were used for bootstrap analysis. Branch support values were highlighted in red and displayed in %.

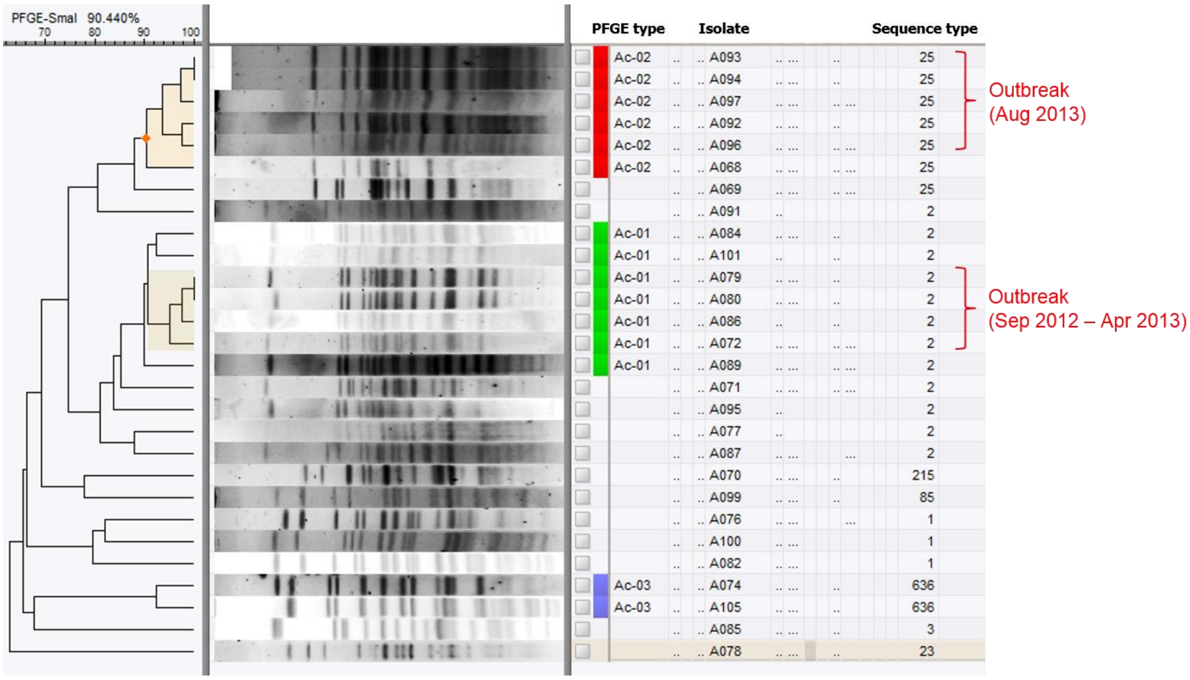

FIGURE S2. Molecular epidemiology of the isolates using pulsed field gel electrophoresis (PFGE). PFGE was performed on the *ApaI*-digested genomic DNA obtained from 28 clinical isolates of *Acinetobacter baumannii*. A dendrogram was generated using the BioNumerics software (version 7.1) based on similarities among the patterns calculated by the Dice coefficient method. A PFGE type was defined by a cluster of isolates showing ≥ 90% similarity or less than 3 band differences. A column of the corresponding sequence types of the isolates was added for comparison. Red right braces were used to highlight the isolates involved in outbreaks of infections.

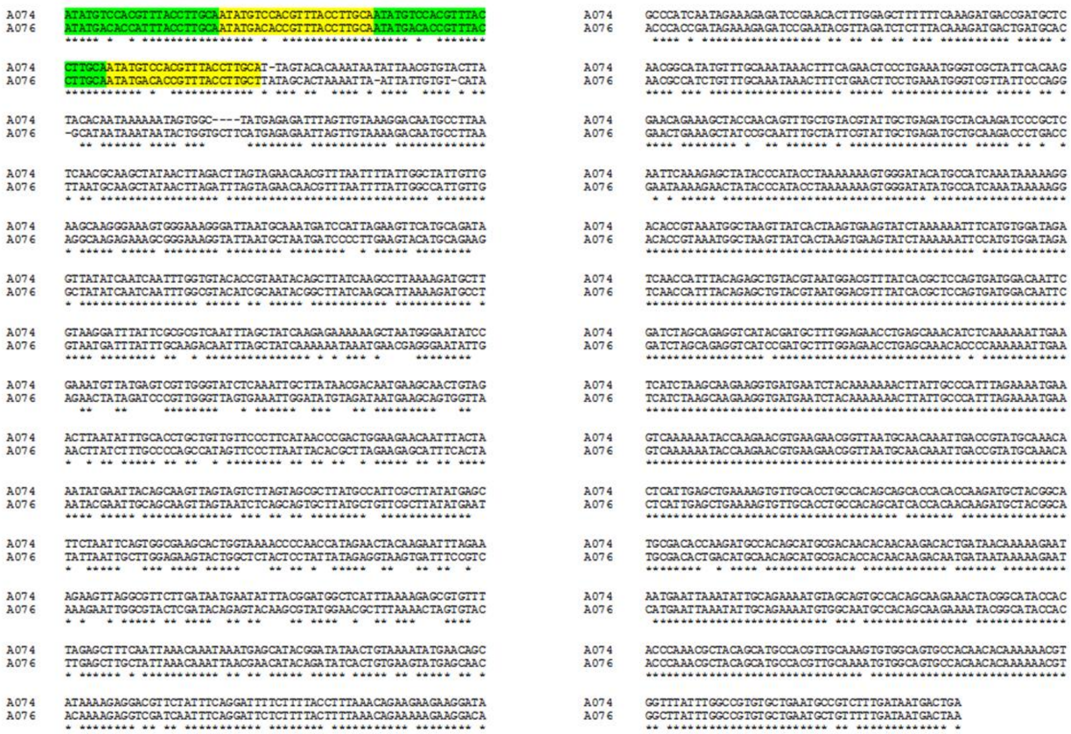

FIGURE S3. Nucleotide sequence comparisons of the replication region of *bla*$_{OXA-24}$- and *bla*$_{OXA-72}$-positive plasmids. The alignment was performed using CLUSTALW (http://www.genome.jp/tools-bin/clustalw) on a region of 1660 bp, carrying 4 iterons (marked in green and yellow) and the *repA* and *repB* genes, from two representative *Acinetobacter baumannii* isolates A074 and A076.

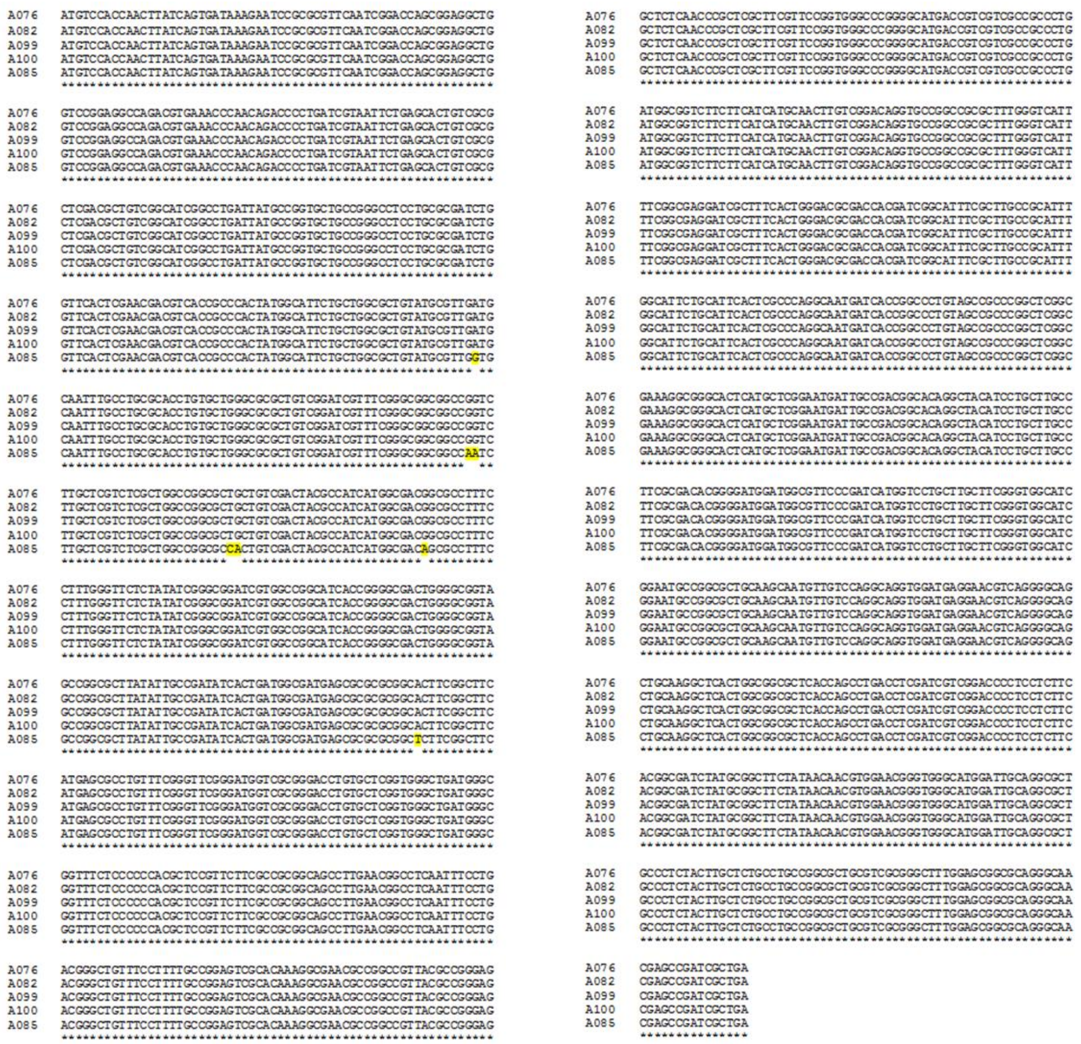

FIGURE S4. Nucleotide sequence comparisons of the *tetA* genes. The alignment was performed using CLUSTALW (http://www.genome.jp/tools-bin/clustalw) on a region of 1275 bp representing the complete sequence of *tetA* identified in isolates A076, A082, A099, A100, and A105. Polymorphisms were highlighted in yellow.

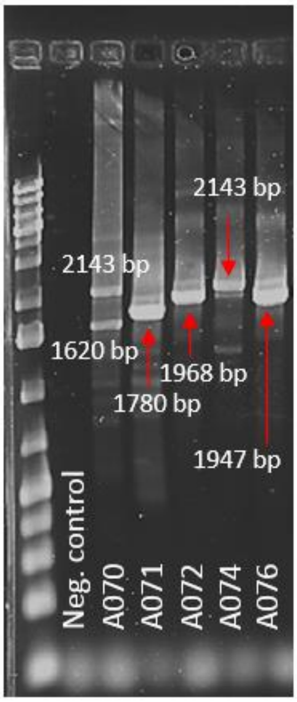

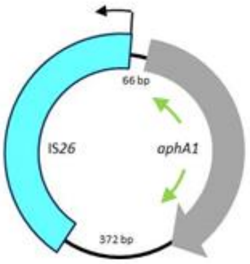

Type and size (bp) of the assembly	Size of the corresponding circular form (bp)	Size of corresponding amplicon (bp)	Representitive isolate
Tn*6020a-1* (2894)	2074	1968	A072
Tn*6020a-2* (3069)	2249	2143 (the upper band in A070)	A070 and A074
Tn*6020a-3* (2873)	2053	1947	A076
Tn*6020b-1* (2706)	1886	1780	A071
Tn*6020b-2* (2546)	1726	1620 (the lower band in A070)	A070

FIGURE S5. Gel electrophoresis of amplicons generated by PCR71. The PCR assay (Table S1) was performed using two *aphA1b* internal outwards primers, shown as green arrows in the graphic map to the left. The size of bands corresponded to the size of Tn*6020*-derived circular forms and to the type and size of Tn*6020* assemblies, as described in the table at the bottom.
